# Coronary artery disease risk gene *PRDM16* regulates smooth muscle homeostasis

**DOI:** 10.1016/j.yjmcc.2026.02.002

**Published:** 2026-02-08

**Authors:** Kunzhe Dong, Yingbing Zuo, Yali Yao, Xiangqin He, Guoqing Hu, Xiaoping Peng, Jiliang Zhou

**Affiliations:** a Immunology Center of Georgia, Medical College of Georgia, Augusta University, Augusta, GA 30912, USA; b Department of Pharmacology & Toxicology, Medical College of Georgia, Augusta University, Augusta, GA 30912, USA; c Department of Cardiology, The First Affiliated Hospital, Jiangxi Medical College, Nanchang University, Nanchang, Jiangxi 330006, China; d Department of Pathology and Translational Pathobiology, Louisiana State University Health Shreveport, Shreveport, LA 71103, USA; e Department of Pharmacology, Toxicology and Neuroscience, Louisiana State University Health Shreveport, Shreveport, LA 71103, USA; f Jiangxi Hypertension Research Institute, Nanchang, Jiangxi 330006, China; g Jiangxi Key Laboratory of Neurological Diseases, Department of Cardiology, The First Affiliated Hospital, Jiangxi Medical College, Nanchang University, Nanchang, Jiangxi 330006, China

**Keywords:** Smooth muscle cells, PRDM16, Neointima formation, Super enhancers, Coronary artery disease

## Abstract

**Objective:**

Vascular smooth muscle cells (VSMCs) are the primary contractile component of blood vessels and can undergo phenotypic switching from a contractile to a synthetic phenotype in vascular diseases such as coronary artery disease (CAD) and restenosis. This process leads to decreased expression of SMC lineage genes and increased proliferative, migratory and secretory abilities that drive disease progression. Super-enhancers (SE) and lineage-specific transcription factors are believed to drive expression of genes that maintain cell identity and homeostasis. The goal of this study is to identify novel regulators of VSMC homeostasis by screening for SE-regulated transcription factors in arterial tissues.

**Approach and results:**

We characterized human artery SEs by analyzing the enhancer histone mark H3K27ac ChIP-seq data of multiple arterial tissues. We unexpectedly discovered the transcription factor PRDM16, a GWAS-identified CAD risk gene with previously well-documented roles in brown adipocytes but with an unknown function in vascular disease progression, is enriched with artery-specific SEs. Further analysis of public bulk RNA-seq and scRNA-seq datasets, as well as qRT-PCR and Western blotting analysis, demonstrated that PRDM16 is highly expressed in arterial tissues and in contractile VSMCs but not in visceral SMCs, and down-regulated in phenotypically modulated VSMCs. To explore the function of *Prdm16* in vivo, we generated both inducible and constitutive *Prdm16* SMC-specific knockout mice and performed bulk RNA-Seq analysis of aortic tissues and left carotid artery ligation to assess neointima formation. SMC-deficiency of *Prdm16* does not affect the aortic morphology at baseline but significantly alters expression of many genes involved in VSMC homeostasis and cardiovascular disease, and suppresses VSMC proliferation and neointima formation in male mice. Specifically, *Prdm16* negatively regulates the expression of *Tgfb2* that encodes an upstream ligand of the TGF-β signaling pathway, by suppressing its promoter activity.

**Conclusions:**

Our results suggest that the CAD risk gene *PRDM16* is highly expressed in VSMCs and is a novel regulator of VSMC homeostasis and neointima formation.

## 1. Introduction

Smooth muscle cells (SMCs) are the major contractile components in hollow organs such as blood vessels and gastrointestinal (GI) tissues. Under normal conditions, SMCs are highly differentiated and contractile, characterized by expressing a unique repertoire of contractile genes [1]. However, under pathological conditions such as coronary artery disease (CAD), SMCs switch their phenotype from a contractile to a synthetic state, as characterized by decreased expression of SMC-specific lineage genes, increased expression of genes associated with proliferation, migration and secretion [1]. This process is known as SMC phenotypic modulation and plays a major role in driving vascular disease progression such as atherosclerosis and restenosis [2]. Despite its importance, the mechanism governing SMC phenotypic modulation is largely unknown. Identifying novel players that maintain SMC identity and homeostasis is an essential step toward understanding the etiology of vascular diseases and developing better clinical approaches.

Super-enhancers (SEs), defined as large clusters of dense enhancers within ~12.5 kb of each other and characterized by exceptionally high transcription factor (TF) and co-activator occupancy [3], are predominant determinants of cell identity and homeostasis by precisely controlling the spatiotemporal expression of cell lineage-specific genes [4–6]. Recent studies have shown that SEs also drive the expression of many critical cardiac lineage-defining TFs, such as TBX3 [7], GATA4 [8], MESP1/2 [9] and HAND2 [10]. Moreover, sequence mutations occurring within the enhancer regions can alter the expression of cardiac genes and cause cardiac dysfunction [11–16]. These studies highlight the potential of identifying novel key regulators that maintain SMC identity and homeostasis by screening for vascular SMC (VSMC)-specific SE-regulated TFs.

PRDM16 is a member of the PR domain-containing (PRDM) family and encodes a zinc-finger TF [17]. It is historically known as a master mediator of brown fat differentiation and identify [18–20]. Interestingly, mutations of *PRDM16* gene have been associated with various cardiovascular diseases including CAD [21], blood pressure-related disorders [22,23], stroke [24] and dilated cardiomyopathy [25,26]. Recent studies have shown that PRDM16 plays a critical role in maintaining myocardial cardiomyocyte identity [27] and regulating arterial flow recovery by maintaining endothelial function [28]. However, further efforts are needed to better understand its in vivo expression pattern [29] and functional role in VSMC-driven vascular diseases [30,31].

Through an unbiased genome-wide screening of multiple public epigenetic and transcriptomic datasets, here we unexpectedly discovered that *PRDM16* is associated with artery-specific SEs and enriched in arterial tissues and VSMCs. To investigate its function, we generated SMC-specific *Prdm16* KO mice and performed whole transcriptome analysis as well as left carotid artery (LCA) ligation to assess neointima formation. Our results show that loss of *Prdm16* in SMCs does not appear to affect aortic morphology at baseline but significantly alters expression of genes involved in VSMC homeostasis, and suppresses VSMC proliferation and neointima formation. Furthermore, our findings suggest that PRDM16 negatively regulates *Tgfb2* in VSMCs by suppressing its promoter activity.

## 2. Materials and methods

The authors declare that all supporting data are included in the article and its online Supplemental Material. The bulk RNA-Seq data generated in this study have been deposited at GEO (Gene Expression Omnibus) database under accession number GSE227692. All animal procedures were performed in accordance with the National Institutes of Health Guide for the Care and Use of Laboratory Animals and approved by the Institutional Animal Care and Use Committee of Augusta University.

## 3. Results

### 3.1. PRDM16 is predominately expressed in arterial tissues and VSMCs

**Fig. 1.**
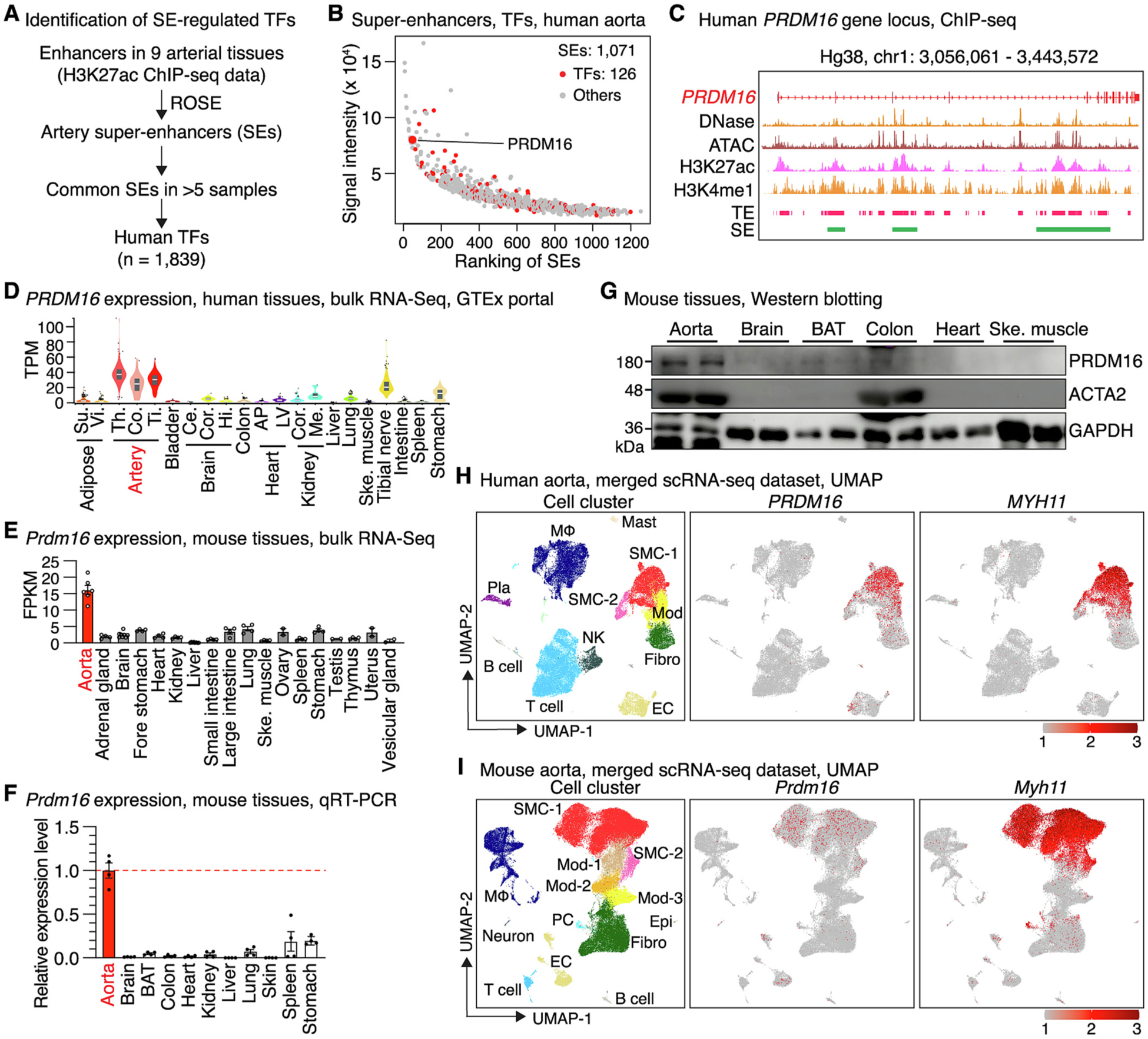
PRDM16 is predominately expressed in arterial tissues and VSMCs. (A) Workflow for identifying super-enhancer (SE)-regulated transcription factors (TF) in human arterial tissues. (B) Ranking of the 1071 arterial SEs. The ranking number and signaling intensity are averaged from the 9 analyzed samples. SEs annotated to transcription factors are highlighted in red and PRDM16 is labeled. (C) Integrative genomics viewer (IGV) tracks of DNase-seq, ATAC-seq, H3K27ac and H3K4me1 ChIP-seq, typical enhancers (TEs) and SEs identified at *PRDM16* gene locus in human aortic tissues. (D-E) *PRDM16* expression across different human (D) and mouse tissues (E) as revealed by GTEx portal and bulk RNA-seq analysis, respectively. Su.: subcutaneous; Vi.: visceral; Th.: thoracic; Co.: coronary; Ti.: tibial; Ce.: cere-bellum; Cor.: cortex; Hi.: Hippocampus; AP: atrial appendage; LV: left ventricle; Me.: medulla; Ske.: skeletal. TPM: transcript per million; FPKM: fragments per kilobase of exon per million mapped fragments. (F-G) *Prdm16* expression across different mouse tissues by (F) qRT-PCR (4 male mice) and (G) Western blotting analysis (2 male mice). Error bars represent mean ± SEM. BAT: brown adipocyte tissue. (H-I) UMAP of cell cluster, expression of *PRDM16* and SMC marker *MYH11* revealed by an integrative scRNA-seq dataset of human (H), and mouse aortic tissues (I) generated by multiple independent studies. Mod: modulated SMCs; Fibro: fibroblast; EC: endothelial cell; PC: pericyte; MΦ: macrophage; NK: natural killer cell; Pla: plasma cell.

To establish the epigenetic regulatory atlas of human blood vessels, we de novo analyzed the enhancer data of 9 human arterial tissues defined by H3K27ac ChIP-seq from ENCODE database (Online Table S1). We identified human artery SEs using the well-established ROSA algorithm [4] and integrated the highly reliable SEs (present in more than 5 samples) with human TF database to screen for SE-regulated TFs in arteries (Fig. 1A). We obtained and prioritized 1071 arterial SEs based on a combination of average ranking and intensity value (Fig. 1B). This analysis revealed 126 SE-regulated TFs in human arterial tissues. Among them, PRDM16, which is a GWAS identified risk gene for multiple cardiovascular diseases with poorly understood function in the progression of vascular disease [21,29], stood out as having the smallest average ranking number and strong intensity across the 9 samples (Fig. 1B).

A closer examination of the human *PRDM16* gene locus revealed an enrichment of artery DNase I hypersensitivity signals and ATAC-seq peaks. In combination with analysis of H3K27ac and H3K4me ChIP datasets, we found *PRDM16* gene locus is enriched with typical enhancers (TEs) and three large SEs identified by ROSE [4] in human arterial tissues (Fig. 1C), indicating a highly accessible chromatin landscape. Consistently, data from GTEx portal database showed that human *PRDM16* expression is highly enriched in all 3 arterial tissues including thoracic, coronary and tibial artery. *PRDM16* expression in the heart is low, although in which the function of PRDM16 has been previously reported [27] (Fig. 1D). Similarly, analysis of bulk RNA-seq of different mouse tissues revealed that *Prdm16* is most abundantly expressed in aortic tissues (Fig. 1E). This aortic tissue-enriched expression pattern of *Prdm16* was further validated by qRT-PCR (Fig. 1F) and Western blotting analysis (Fig. 1G and Online Fig. S1) that included the brown adipocyte tissue (BAT), a tissue in which PRDM16 function was best documented [18–20]. Notably, unlike the pan-SMC marker ACTA2, PRDM16 is minimally expressed in the colon, a visceral SMC-rich tissue. These results collectively indicate that PRDM16 expression is enriched in arterial tissues in both human and mouse.

To further define the cell types that express *PRDM16* in blood vessels, we examined its expression in the integrative scRNA-seq datasets of human and mouse aortic tissues, respectively. Both datasets recapitulated major vascular cell types such as normal SMCs (SMC), phenotypically modulated SMC clusters (Mod), fibroblast (Fibro), endothelial cells (EC), and immune cells including T cells, B cells, macrophages (MΦ) and natural killer (NK) cells (Fig. 1H–I), as defined by highly expressed cell type-specific marker genes (Online Fig. S2). This in silico analysis revealed that *PRDM16* is predominantly expressed in normal contractile SMCs and to a lesser extent in ECs, while down-regulated in phenotypically modulated SMCs (Fig. 1H–I). PRDM16 has recently been reported to play a critical role in cardiomyocytes [27,32–34]. To further characterize its expression in the heart, we re-analyzed publicly available scRNA-seq data generated in embryonic and adult heart of both human (Online Fig. S3) and mouse (Online Fig. S4). These analyses revealed the *PRDM16* expression is present in SMCs, ECs, cardiomyocytes and adipocytes in heart of both human and mouse, without the VSMC-enriched pattern observed in arterial tissues. *Re*-analysis of scRNA-seq of SMC-enriched intestinal tissues from both human and mouse revealed that *PRDM16* expression is negligible in visceral SMCs (Online Fig. S5). Taken together, these findings suggest that the CAD gene PRDM16 is highly expressed in arterial tissues and VSMCs but not in visceral SMCs, suggesting a previously unrecognized role for PRDM16 in VSMCs.

### 3.2. SMC-specific Prdm16 deletion alters VSMC transcriptomes in mouse aorta

**Fig. 2.**
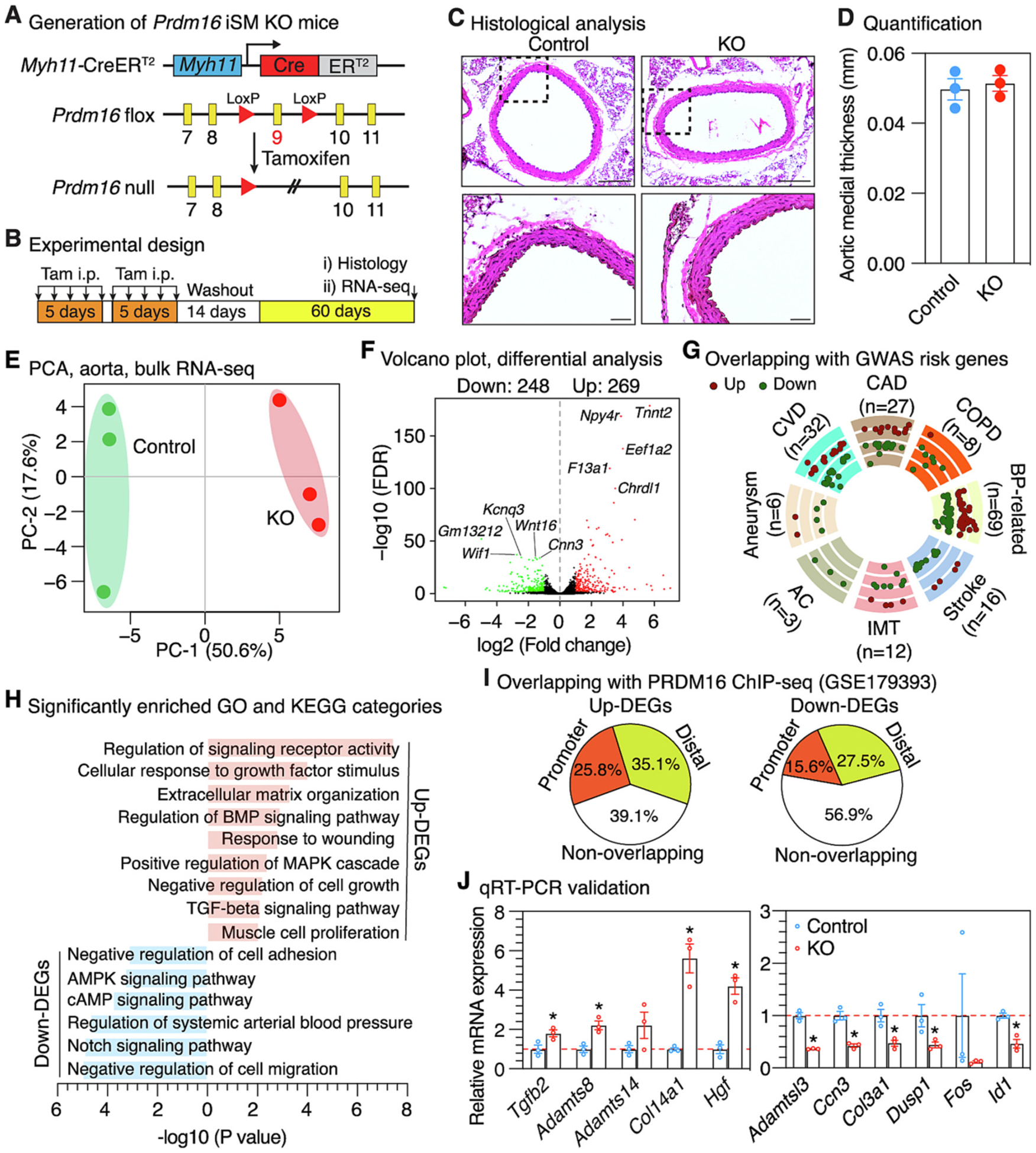
SMC-specific *Prdm16* deletion in mice alters transcriptome of VSMCs. (A) Strategy for generating inducible smooth muscle-specific *Prdm16* KO mice (iSM KO). (B) Schematic diagram of experimental design. Tam: Tamoxifen; i.p.: intraperitoneal injection. (C) Representative pictures of H&E staining on thoracic aorta from control and *Prdm16* iSM KO mice. The boxed areas are magnified beneath. Scale bar: 50 μm. (D) Quantification of the aortic medial thickness. Error bars represent mean ± SEM. *N* = 3 for both control and KO group. (E) Principal component analysis (PCA) using all the expressed genes identified by bulk RNA-seq. (F) Volcano plot showing the differentially expressed genes (fold change >2 and FDR <0.05) identified by bulk RNA-seq in aortic tissues of *Prdm16* iSM KO mice as compared to control mice. The top 5 most significantly changed up- and down-regulated genes are labeled, respectively. (G) Circle plot showing the overlapping dysregulated genes identified by bulk RNA-seq following *Prdm16* deletion with GWAS risk genes of various cardiovascular diseases. Each dot represents an individual gene, with red and green dots indicating up- and down-regulated genes, respectively. The radial position of the dots reflects the magnitude and direction of differential expression based on the fold change. Dots closer to the center indicate genes with greater down-regulation, whereas those toward the outer rings indicate genes with stronger up-regulation. CAD: coronary artery disease; COPD: chronic occlusive pulmonary disease; BP-related: blood pressure-related disorders; IMT: intimal-media thickness; AC: arterial calcification; CVD: cardiovascular disease. (H) Selected enriched gene ontology (GO) terms and KEGG pathways for up- and down-regulated genes identified by bulk RNA-seq following *Prdm16* deletion. (I) Genomic distribution of common PRDM16-dependent genes identified by bulk RNA-seq in *Prdm16* iSM KO mouse aorta and a public PRDM16 ChIP-seq data generated in mouse heart. (J) qRT-PCR validation of selected differentially expressed genes identified by RNA-Seq. Error bars represent mean ± SEM. *N* = 3 for both control and KO group. *P < 0.05; unpaired Student’s *t*-test.

As an initial step to explore the VSMC function of PRDM16 in vivo, we generated inducible SMC-specific *Prdm16* KO mice (iSM KO) by crossing *Prdm16*^*F/F*^ mice [18] with *Myh11*-CreER^T2^ mice [35] (Fig. 2A). Upon tamoxifen administration, Cre activation led to the excision of floxed exon 9, introducing a frameshift mutation and producing *Prdm16* null alleles in SMCs. Adult mice were injected with tamoxifen to initiate Cre activation, followed by 2 weeks of washout period, and aortic tissues were harvested for histological and bulk RNA-seq analysis after additional 60 days (Fig. 2B). The specific and efficient deletion of *Prdm16* exon 9 in the arteries of *Prdm16* iSM KO mice was validated by detection of the deleted allele with PCR genotyping and was further confirmed by qRT-PCR analysis (Online Fig. S6A–C). Mice lacking *Prdm16* in SMCs exhibit no obvious gross abnormalities and have comparable body weight to control mice (Online Fig. S6D). As an initial screen for potential morphological changes in the aorta, we performed H&E staining on thoracic aorta. This analysis revealed no significant changes in medial thickness (Fig. 2C–D) and lumen area of aortas from *Prdm16* iSM KO mice compared to control mice (Online Fig. S6E).

We next sought to assess the effect of *Prdm16* loss on the gene expression programs of VSMCs. We performed bulk RNA-Seq on aortic tissues from both *Prdm16* iSM KO and control mice 60 days after tamoxifen administration, allowing sufficient time to avoid acute tamoxifen- or Cre-related effects and capture stable transcriptional changes driven by *Prdm16* deficiency. As anticipated, examination of RNA-seq reads demonstrated that the number of reads derived from the floxed exon 9 are dramatically reduced in *Prdm16* iSM KO mouse aorta as compared to that of control mice (Online Fig. 6F). PCA analysis using all the detected expressed genes revealed KO samples were clearly segregated from control samples (Fig. 2E), indicating altered global transcriptomic profiles in the absence of *Prdm16*. Differential analysis revealed a total of 269 and 248 up- and down-regulated genes (Fold change >2 and FDR <0.05) (Fig. 2F and Online Table S4). Interestingly, 43 of these genes have been identified as risk genes by GWAS for various cardiovascular diseases such as CAD, chronic occlusive pulmonary disease (COPD), BP-related disorders and aneurysm (Fig. 2G and Online Table S4). Subsequent functional enrichment analysis revealed differentially expressed genes were significantly overrepresented in a wide spectrum of functional categories with some of them strongly implicated in VSMC phenotypic switching and vascular disease development. For instance, up-regulated genes were involved in response to external stimuli, extracellular matrix organization, MAPK signaling pathway and muscle cell proliferation. Notably, genes involved in BMP and TGF-β signaling pathways were up-regulated in *Prdm16* KO VSMCs (Fig. 2H). Down-regulated genes are mainly associated with regulation of cell adhesion and cell migration, as well as AMPK, cAMP and Notch signaling pathways (Fig. 2H).

To examine whether these dysregulated genes are direct PRDM16 targets or not, we integrated PRDM16 ChIP-seq data generated from embryonic mouse heart [27]. PRDM16 ChIP-seq peaks were found in 60.9% of up-regulated and 43.1% of the down-regulated genes, with 25.8% and 15.6% respectively located within the proximal promoter regions (Fig. 2I and Online Table S4). Furthermore, we selected a list of up–/down-regulated differentially expressed genes including *Tgfb2*, an upstream effector of TGF-β signaling pathway, and matrix genes (*Adamts8*, *Adamts14*, *Adamtsl3*, *Col14a1* and *Col3a1*), as well as several other genes with little known function in SMCs and vascular diseases such as *Hgf*, *Fos* and *Id1* for validation with qRT-PCR (Online Fig. 6G). These selected genes are either risk genes for cardiovascular diseases (Online Fig. 6H) or/and contain PRDM16 ChIP-seq peaks within their genomic region (Online Fig. 6I). The differential expression of most of the selected genes following *Prdm16* KO was confirmed by qRT-PCR analysis (Fig. 2J). Collectively, our results suggest that *Prdm16* deficiency alters VSMC transcriptomes.

### 3.3. SMC-specific Prdm16 deletion suppresses neointima formation in male mice

**Fig. 3.**
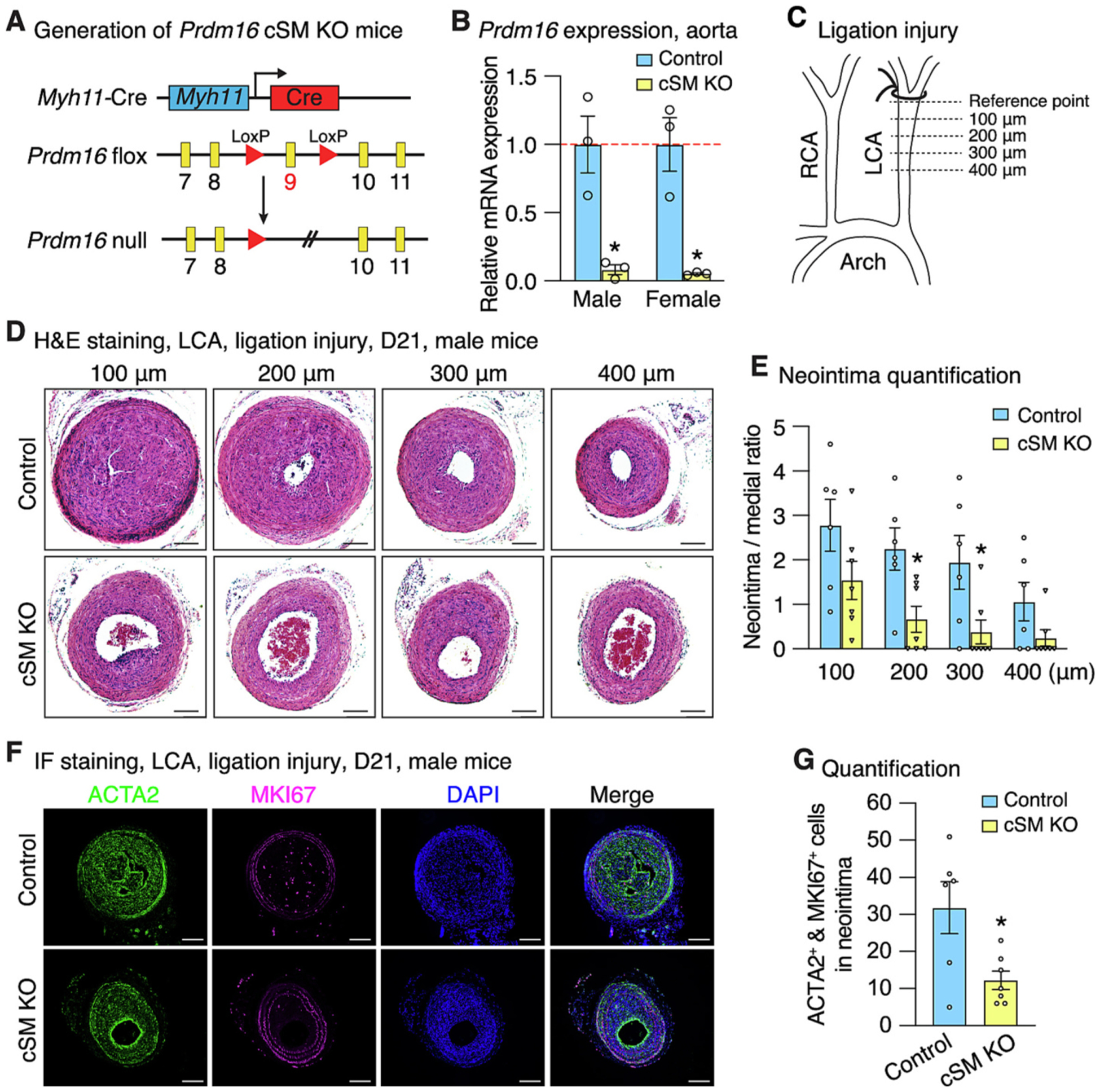
SMC-specific *Prdm16* deletion suppresses neointima formation in male mice. (A) Strategy to generate constitutive SMC-specific *Prdm16* KO mice (cSM KO). (B) qRT-PCR analysis of *Prdm16* expression in aortas from male and female *Prdm16* cSM KO mice and control mice. Error bars represent mean ± SEM. N = 3 for both control and *Prdm16* cSM KO group; *P < 0.05; unpaired Student’s *t*-test. (C) Schematic diagram illustrating the left carotid artery (LCA) ligation injury model and corresponding locations collected for histological analysis. RCA: right carotid artery. The reference point located beneath the ligation site was set at a position where the ligature did not distort the vessel and the elastic laminae remained intact. (D) Hematoxylin & eosin (H&E) staining of LCA of control and *Prdm16* cSM KO male mice 21 days (D21) upon ligation injury. Scale bar: 100 μm. (E) Quantification of the neointima-to-media ratio in the injured LCA of male mice at four different positions from the reference point. Error bars represent mean ± SEM. *N* = 6 for control and *N* = 7 for *Prdm16* cSM KO group; *P < 0.05; unpaired Student’s *t*-test. (F) Immunofluorescence staining of MKI67 (purple) and ACTA2 (green) in ligation-injured LCA of control and *Prdm16* cSM KO male mice. Nuclei were counterstained with DAPI (blue). Scale bar: 100 μm. (G) Quantification of numbers of ACTA2 and MKI67 double positive cells in medial and neointimal areas of ligation-injured LCA in control and *Prdm16* cSM KO male mice. Error bars represent mean ± SEM. N = 6 for control and N = 7 for *Prdm16* cSM KO group; *P < 0.05; unpaired Student’s *t*-test.

**Fig. 4.**
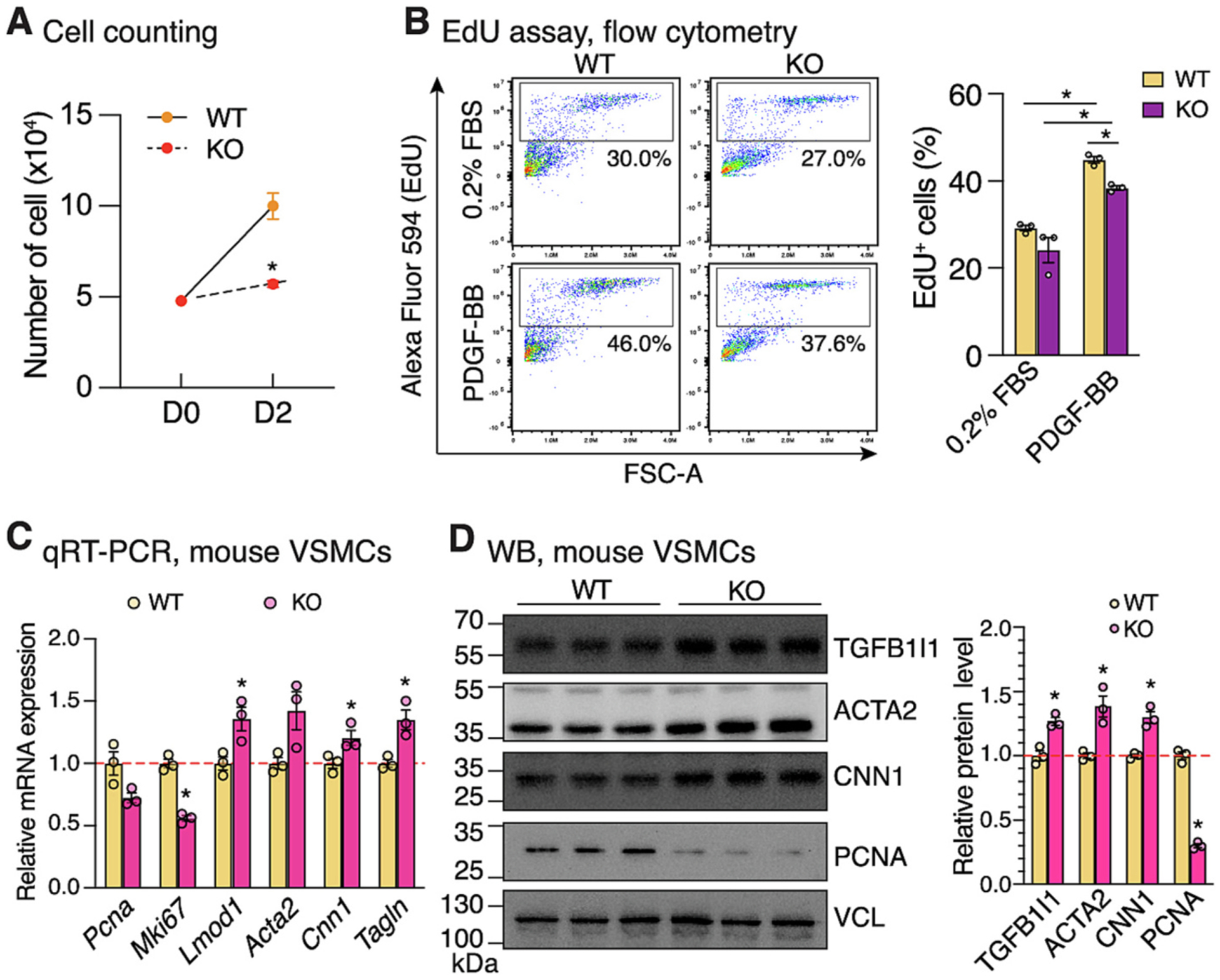
*Prdm16* deficiency inhibits VSMC proliferation in vitro. (A) VSMCs isolated from WT (N = 3) or constitutive SMC-specific *Prdm16* KO mice (cSM KO) (N = 3) were seeded at equal density (4.8 × 10^4^ cells) on Day 0 (D0) and cultured in full medium (10% FBS) for 2 days (D2) for direct cell counting. (B) Flow cytometry analysis of EdU^+^ cells from WT and *Prdm16* KO VSMCs cultured in medium containing 0.2% FBS with or without PDGF-BB stimulation (50 ng/mL) for 24 h. Representative flow plots (left) and quantification (right) are shown. (C) qRT-PCR and (D) Western blotting analyses of proliferation-associated and contractile marker genes in VSMCs cultured in full medium for 2 and 3 days, respectively. Densitometric quantification of Western blotting is shown on the right. For all analyses, error bars represent mean ± SEM. N = 3 for both WT and KO group; *P < 0.05; Unpaired Student’s *t*-test.

Since the inducible *Myh11*-CreER^T2^ driver is Y chromosome-linked and restricted to male mice [36], we next generated a constitutive SMC-specific *Prdm16* KO (cSM KO) mice using a novel *Myh11*-Cre line we recently developed [37] (Fig. 3A). This Cre line constitutively expresses Cre specifically in SMCs beginning in early embryonic development, driven by the endogenous *Myh11* promoter located on chromosome 16, thus enabling investigation of *Prdm16* function in both male and female mice [37]. Deletion of *Prdm16* in the aorta was confirmed by genotyping in both sexes showing the presence of deleted allele specifically in aortic tissues such as brachiocephalic artery but not in tails (Online Fig. S7A). Data from qRT-PCR further supported a significant reduction in *Prdm16* expression in the aorta from both sexes of mice (Fig. 3B). Both male and female KO mice showed no obvious abnormalities and had comparable body weights to control mice (Online Fig. S7B). To examine the in vivo function of PRDM16 under pathological conditions, we performed LCA ligation in male and female *Prdm16* cSM KO and control mice. Injured LCA tissues were collected 21 days post-ligation for neointima assessment at different distances (100, 200, 300 and 400 μm) from a reference point located beneath the ligation site, where the ligature did not distort the vessel and the elastic laminae remained intact (Fig. 3C), as previously described [38,39]. H&E staining revealed significantly reduced neointima formation at 200 and 300 μm in male *Prdm16* cSM KO mice, as evidenced by decreased neointimal area (Online Fig. S7C) and reduced neointima-to-medial ratio (Fig. 3D & E). No significant differences were observed in medial layer area or lumen area, although a trend toward increased lumen area in KO mice was observed (Online Fig. S7C). In contrast, no difference was detected between KO and control female mice (Online Fig. S7D–E). Immunofluorescence staining revealed a reduction in MKI67^+^/ACTA2^+^ cells in injured LCA of male KO mice (Fig. 3F & G), indicating suppressed VSMC proliferation in the absence of *Prdm16* within neointima. To further substantiate this specifically in VSMCs, we isolated primary VSMCs from cSM *Prdm16* KO mice and control aortas, and cultured them under full medium containing 10% Fetal Bovine Serum (FBS) or stimulated with PDGF-BB to mimic proliferative cues during neointima formation following arterial injury. Consistent with the in vivo findings, *Prdm16*-deficient VSMCs exhibited reduced proliferation as determined by direct cell counting (Fig. 4A). EdU incorporation assays followed by flow cytometry (Online Fig. S7F–G) further revealed a marked reduction in EdU^+^ cells in KO compared to WT VSMCs after PDGF-BB stimulation (Fig. 4B). Moreover, qRT-PCR and Western blotting analyses demonstrated markedly decreased expression of proliferation-associated genes such as *Pcna* and *Mki67*, accompanied by increased expression of SMC contractile markers in KO VSMCs (Fig. 4C–D). In addition, siRNA-mediated acute knockdown of *Prdm16* in WT VSMCs produced similar results, showing decreased proliferative gene expression while increasing contractile gene expression (Online Fig. S7H). These results collectively demonstrate that *Prdm16* deletion in SMCs inhibits ligation-induced neointima formation in male mice, likely due to decreased VSMC proliferation.

### 3.4. PRDM16 negatively regulates Tgfb2 expression by suppressing its promoter activity

**Fig. 5.**
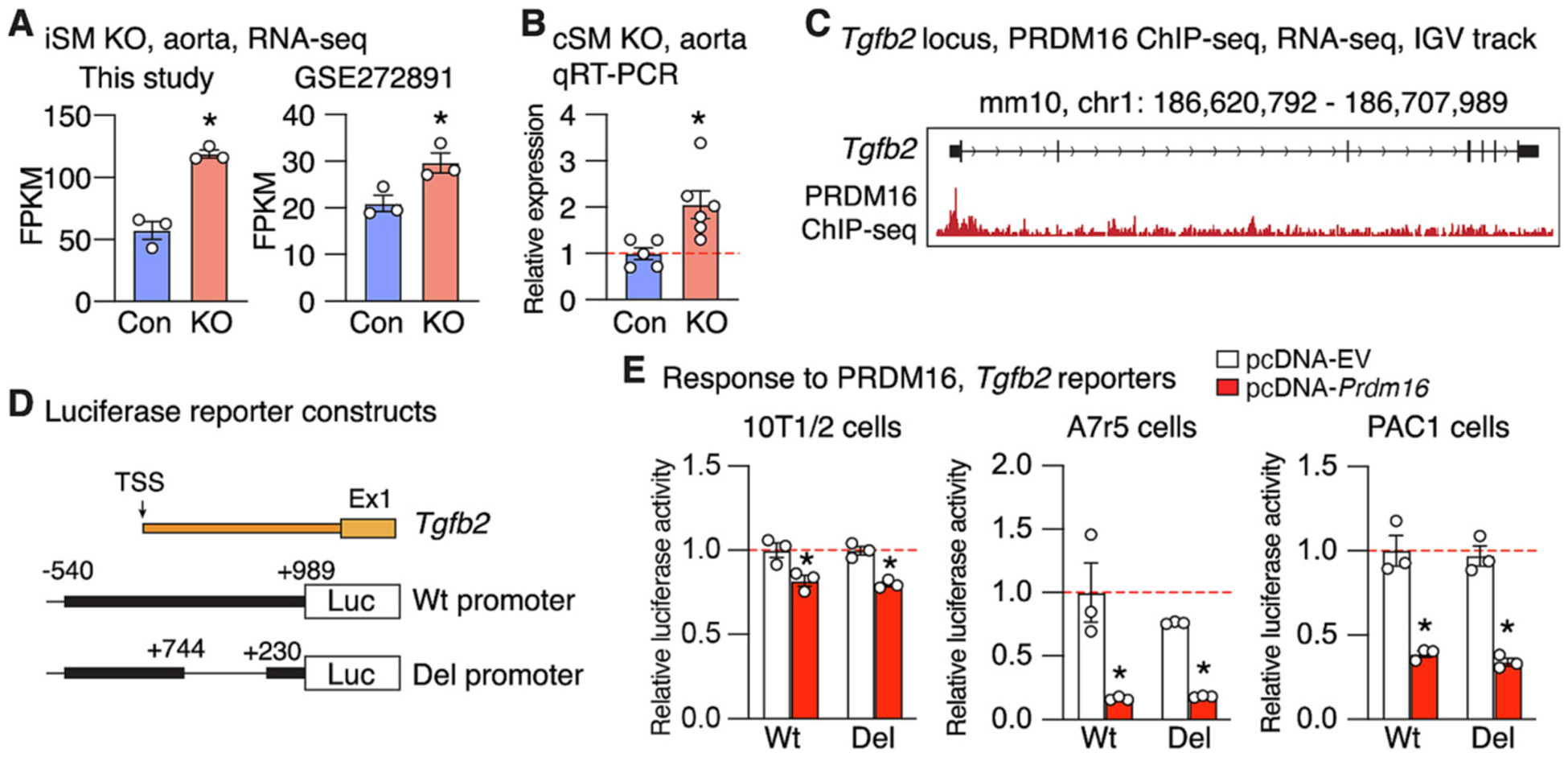
PRDM16 negatively regulates *Tgfb2* expression. (A) *Tgfb2* expression is increased in aortas of *Prdm16* inducible SMC-specific (iSM) KO mice, as revealed by RNA-seq analysis from this study (left) and a previous study (GSE272891, right). FPKM: Fragments Per Kilobase of exon model per Million mapped fragments. Error bars represent mean ± SEM. N = 3 for both control and KO group; *FDR <0.05; Differentiall analysis by DESeq2 R package. (B) Increased *Tgfb2* expression is also observed in aortas of *Prdm16* constitutive SMC-specific (cSM) KO male mice by qRT-PCR analysis. Error bars represent mean ± SEM. *N* = 5 for control and N = 6 for KO group; *P < 0.05; unpaired Student’s *t*-test. (C) IGV tracks of PRDM16 ChIP-seq from a public dataset generated from mouse heart tissues showing robust PRDM16 binding peaks enriched in the promoter region of *Tgfb2* gene. (D) Schematic illustrating the luciferase reporter constructs containing either the WT mouse *Tgfb2* promoter (−540 to +989 bp) (Wt) or a deletion (Del) lacking the putative PRDM16-bound region (+230 to +744 bp) identified by heart ChIP-seq data. Coordinates are relative to the transcription start site (TSS) of *Tgfb2*. Ex1: exon 1. (E) Dual luciferase reporter assays in 10 T1/2 fibroblasts (left), A7r5 (middle) and PAC1 cells (right) 24 h after co-transfection with PRDM16 expression (pcDNA-*Prdm16*) or with empty vector (EV) (pcDNA-EV) as control. Error bars represent mean ± SEM. N = 3 for both Wt and Del group; *P < 0.05; unpaired Student’s *t*-test.

We hypothesized that TGFB2, an upstream effector of TGF-β signaling pathway known to play critical roles in SMC and vascular biology [40], is a downstream target of PRDM16. Our RNA-seq analysis revealed that *Tgfb2* expression is significantly up-regulated in aortas of *Prdm16* iSM KO mice (Fig. 5A, left). Consistently, bulk RNA-seq data from a previous study [31] which used the same Cre driver to delete *Prdm16* in SMCs, also showed increased *Tgfb2* expression in aortic tissues (Fig. 5A, right). Notably, the expression of *Tgfb1* and *Tgfb3*, the other two ligands of the TGF-β pathway, remained unchanged in both datasets (Online Fig. S8A). To further validate this finding, we performed qRT-PCR analysis which confirmed the elevated *Tgfb2* expression in the aortas of *Prdm16* cSM KO mice (Fig. 5B), along with dysregulation of several other genes identified in the RNA-seq analysis of *Prdm16* iSM KO mice (Online Fig. 8B). Interestingly, ChIP-seq data from mouse heart revealed PRDM16 binding peaks at the promoter region of *Tgfb2* (Fig. 5C) [27], suggesting potential direct transcriptional regulation. To test this, we generated mouse *Tgfb2* promoter luciferase reporters containing either the full −540 to +989 promoter fragment (Wt) or a deletion lacking the putative PRDM16-bound region (Del) (Fig. 5D), and assessed their responsiveness to PRDM16 co-expression in 10 T1/2 fibroblasts and two VSMC lines (A7r5 and PAC1). In 10 T1/2 cells, PRDM16 induced a modest but significant suppression of luciferase activity from both Wt and Del reporters (Fig. 5E, left). In contrast, in both VSMC lines, PRDM16 dramatically inhibited *Tgfb2* promoter activity, suggesting that fibroblasts may lack cofactors required for full PRDM16-mediated suppression. Notably, deletion of the putative PRDM16-bound region identified by heart ChIP-seq did not diminish this suppression, as WT and Del reporters responded to PRDM16 similarly (Fig. 5E, middle and right). Together, these results suggest that PRDM16 negatively regulates *Tgfb2* transcription in VSMCs by suppressing its promoter activity, and the heart ChIP-seq-identified PRDM16-bound region (+230 to +744) is not required for this regulation.

## 4. Discussion

In this study, we demonstrate that *PRDM16*, a GWAS identified CAD risk gene with previously well-documented roles in brown adipocytes [18–20] and cardiomyocytes [27], is enriched with artery SEs and highly expressed in arterial tissues and VSMCs, but not in visceral SMCs. Deletion of *Prdm16* gene in SMCs in mice does not affect the aortic morphology at baseline but significantly alters expression of many genes involved in VSMC phenotypic modulation and vascular disease progression. Moreover, *Prdm16* loss in SMCs attenuates cell proliferation and ligation-induced neointima formation in male mice.

PRDM16 is best known as a master molecular switch of brown fat differentiation [18–20] and has also been shown to play critical roles in cardiomyocyte compaction during murine embryonic development [27]. A recent study further demonstrated that adipocyte-specific *Prdm16* deficient mice, characterized by loss of beige adipocyte identify, develop marked vascular remodeling and elevated blood pressure, underscoring a broader role for PRDM16 in vascular homeostasis [41]. In this study, we performed a comprehensive analysis of *PRDM16* expression and demonstrated that *PRDM16* gene locus harbors numerous artery enhancers and SEs, and is most abundantly expressed in arterial tissues compared to other tissues, including the brown adipocyte tissue and heart (Fig. 1A–G). Among vascular cell types, *PRDM16* expression is highly enriched in SMCs as revealed by scRNA-seq of human and mouse aortic tissues (Fig. 1H–I). This finding is supported by recent scATAC-seq (single-cell sequencing assay for transposase-accessible chromatin) studies of human aortic tissues showing SMC-specific chromatin accessibility at the *PRDM16* locus [42,43], along with other recent reports [30,31,44]. PRDM16 is also expressed in ECs with a much lower abundance as compared to SMCs, in agreement with the recent study reporting that PRDM16 plays a role in regulating arterial flow recovery via endothelial function [28]. Interestingly, unlike other SMC-enriched genes that are expressed in both vascular and visceral SMCs of GI tissues such as MYOCD [45], LMOD1 [46] and *CARMN* [47], PRDM16 exhibits negligible expression in GI tissues and visceral SMCs (Fig. 1D–G & Online Fig. S3–5). Previous studies showed that global or SMC-specific knockout of these pan SMC-enriched genes in mice often leads to lethal GI phenotypes [48–50], which largely impedes the efforts for investigating their vascular function. Therefore, the VSMC-enriched expression of PRDM16 offers a unique opportunity to study its function in VSMCs and vascular disease by inherently avoiding GI confounders. The regulatory mechanism conferring the VSMC-enriched expression of PRDM16 is unclear. We speculate it lies in chromatin landscape, as the gene locus contains many chromatin regions that are specifically accessible in VSMCs [42,43]. Future studies may focus on unveiling which regulatory element(s) and upstream transcription factor(s) direct the enriched expression of PRDM16 in VSMCs.

To determine the consequence of loss of PRDM16 in SMCs in vivo, we deleted *Prdm16* specifically in SMCs in mice. Our results show that *Prdm16* deficiency in adult SMCs does not affect the aortic morphology at baseline (Fig. 2C–D) but significantly disrupted the transcriptomic profiles (Fig. 2E–F). The dysregulated genes upon *Prdm16* ablation, which may reflect both direct transcriptional targets of PRDM16 as well as secondary downstream effects, include many known risk genes for various cardiovascular disorders (Fig. 2G), suggesting PRDM16 is a broader regulator of cardiovascular diseases-associated genes. These transcriptomic changes likely disrupt VSMC homeostasis and contribute to vascular disease development. Supporting this, recent studies have shown that SMC-specific *Prdm16* KO mice exhibited aberrant circadian blood pressure rhythms [30], increased abdominal aortic aneurysm development [31], and formation of collagen-rich atherosclerotic plaques with thick fibrous caps and few foam cells [44]. In our study, we further explored its role in neointima formation, a VSMC proliferation-driven pathology modeling arterial restenosis [51]. Unexpectedly, we found that *Prdm16* deficiency attenuates ligation-induced neointima formation in male mice and inhibits VSMC proliferation. Given that PRDM16 expression is enriched in contractile SMCs and downregulated during SMC phenotypic modulation, this paradoxical finding suggests that PRDM16 exerts a more complex, context-dependent role beyond maintaining VSMC homeostasis under baseline conditions. Specifically, it may facilitate pro-remodeling or proliferative gene programs in response to injury. Interestingly, this inhibitory effect on neointima formation was not observed in female mice, indicating a sex-dependent response. Although the mechanisms underlying this difference remain unclear, prior studies support sex-dependent roles of PRDM16 in cardiovascular biology. For example, cardiac-specific *Prdm16* deletion results in sex-dependent cardiomyopathy and increased cardiac mortality, with greater susceptibility in females [52]. In contrast, SMC-specific deletion of *Prdm16* has been reported to produce similar hypotensive phenotypes in both male and female mice [30], suggesting that the sex differences in PRDM16 function may depend on the disease context. It will therefore be important for future studies to determine whether PRDM16 exerts sex-specific effects in other vascular disease models such as atherosclerosis [53] and aneurysm [31], which have not been systematically examined. In addition, androgen receptor signaling has been shown to negatively regulate PRDM16 expression in beige adipocyte [54], raising the possibility that hormonal pathways may similarly modulate PRDM16 activity in VSMCs, a question that warrants further investigation. Overall, these findings position PRDM16 as a major regulator of VSMC function and a potential therapeutic target in diverse vascular diseases.

Among the genes upregulated following *Prdm16* deletion is *Tgfb2*, which encodes TGFB2, an upstream activator of TGF-β signaling pathway and is a GWAS identified risk gene for multiple vascular diseases such as aortic aneurysm [55,56], BP-related disorders [57], and COPD [58]. Elevated *Tgfb2* expression is consistently observed in both iSM and cSM *Prdm16* KO aortas, which is further corroborated by a previous study using the iSM KO model [31] (Fig. 5A & B). Similarly, increased *Tgfb2* expression has also been observed following *Prdm16* deletion in cardiomyocytes across multiple independent studies [27,33,34]. This cross-reference validation provides strong evidence that PRDM16 negatively regulates *Tgfb2* transcription. ChIP-seq from mouse heart tissue [27] revealed the presence of PRDM16 binding peaks within its proximal promoter region. However, our luciferase reporter assays indicate that PRDM16 suppresses the *Tgfb2* promoter activity in VSMCs independently of this putative binding site (Fig. 5D–E), suggesting that PRDM16 may regulate the same target gene via distinct regulatory regions in a cell type-dependent manner. Furthermore, because the inhibitory effect was notably weaker in fibroblast relative to VSMCs (Fig. 5E), we reasoned that PRDM16 may require VSMC-expressing co-factors to exert its repressive function on *Tgfb2*, consistent with its role as a transactional co-regulator [17]. TGF-β consists of 3 ligand members including TGFB1, TGFB2 and TGFB3. Our results support that PRDM16 selectively regulates TGFB2, without affecting the expression of the other two. It remains unclear whether the attenuated neointima formation in *Prdm16* SMC KO male mice is a direct consequence of elevated *Tgfb2* expression. TGF-β signaling pathway plays diverse, context-dependent roles in regulating VSMC phenotype and function [40]. While many studies have shown that TGF-β signaling pathway is activated in injured arteries and promotes injury-induced neointima formation [59–62], others suggest it can inhibit VSMC proliferation and migration [63]. Furthermore, most of these studies have focused on TGFB1, whereas the specific role of TGFB2 remains less well understood. Notably, TGFB2 and TGFB1 are not functionally redundant, as their knockout mice show distinct phenotypes [64]. Specifically, *Tgfb2* KO mice exhibit perinatal lethality with smaller ascending aortas and thinning of aortic wall, and other developmental defects in heart, lung, inner ear, limb, spinal column, and urogenital defects [65], while *Tgfb1* null mice develop immune system defects with an inflammatory phenotype [66]. Additionally, TGFB2 haploinsufficiency has been shown to impair SMC differentiation [67] and predispose to thoracic aortic aneurysms [55]. Consistent with this, deletion of SMC-specific *Tgfb2* in adult mice leads to vascular wall medial degeneration, aneurysm formation, dissection, and rupture [68]. These findings collectively support a role for TGFB2 in maintaining VSMC differentiation phenotypes, which typically shift toward a synthetic and proliferative state in response to injury. Therefore, our observations that *Prdm16* deletion leads to *Tgfb2* upregulation while concurrently suppressing neointima formation and VSMC proliferation raises the possibility that TGFB2 may function to inhibit VSMC phenotypic switching under certain conditions.

In summary, our study identifies that *PRDM16*, a CAD-associated gene, is highly expressed in aortic tissues and VSMCs, and establishes it as a novel regulator of VSMC homeostasis. In addition to its recently reported roles in aneurysm [31], blood pressure regulation [30] and atherosclerosis [44], we provide new insights into the in vivo function of PRDM16 in vascular remodeling following injury. Targeting PRDM16 expression in VSMCs may represent a promising therapeutic approach for treating vascular diseases.

## Supplementary Material

1

2

## Appendix A. Supplementary data

Supplementary data to this article can be found online at https://doi.org/10.1016/j.yjmcc.2026.02.002.
