# Supplementary material for: Coronary artery disease risk gene PRDM16 regulates smooth muscle homeostasis
Source: J Mol Cell Cardiol. Author manuscript; Available in PMC 2026 May 27. (PMC13215035; doi:10.1016/j.yjmcc.2026.02.002)
Supplement: 1 [file NIHMS2177815-supplement-1.docx]

**SUPPLEMENTAL MATERIALS**

**Coronary Artery Disease Risk Gene *PRDM16* Regulates Smooth Muscle Homeostasis**

Kunzhe Dong, Yingbing Zuo, Yali Yao, Xiangqin He, Guoqing Hu, Xiaoping Peng, Jiliang Zhou

**^#^ Co-corresponding author:**

**Kunzhe Dong, Ph.D.**

Immunology Center of Georgia

Department of Pharmacology & Toxicology

Medical College of Georgia

Augusta University

1462 Laney Walker Blvd., Augusta GA 30907

Email: [kdong@augusta.edu](mailto:kdong@augusta.edu)

**Jiliang Zhou, M.D./Ph.D.**

Department of Pharmacology, Toxicology & Neuroscience

Louisiana State University Health Shreveport

1501 Kings Highway, Shreveport, LA 71103

Office Phone #: 318-675-7852

Email: [jiliang.zhou@lsuhs.edu](mailto:jiliang.zhou@lsuhs.edu)

**Online Methods**

**Identification of human artery SEs**

The aligned Bam and narrowPeak files of H3K27ac ChIP-seq data generated from 9 different human arterial tissues were downloaded from ENCODE database (**Online Table S1**) and used to identify artery SEs by using ROSE program [1] with the default arguments. Super-imposed SEs that are present in at least 5 artery samples were selected and further ranked based on a combination of average ranking and intensity across the 9 samples. For each SE, the associated genes were annotated using R package ChIPseeker [2] and intersected with 1,839 human transcription factors obtained from SCENIC database [3].

**Bioinformatics analysis of public bulk RNA-seq and scRNA-seq data**

Bulk RNA-seq data of aortic tissue [4] and 10 other tissues [5] of mouse were used to evaluate *Prdm16* abundance indicated by FPKM (fragments per kilobase of exon per million mapped fragments) across different mouse tissues. Raw Fastq files of bulk RNA-seq data from WT and *Prdm16* inducible SMC-specific KO aortas from a previous study [6] were downloaded (GEO272791) and processed as previously described [7]. Briefly, raw reads were first trimmed for quality control using Trimmomatic 0.38 [8] and then aligned to mm10 reference genome using STAR aligner [9]. Gene-level raw counts were quantified by featureCounts 1.6.2 [10] and differential analysis was performed using R package DESeq2 [11].

To examine *PRDM16* expression at the single-cell level, we generated a merged scRNA-seq dataset of aortic tissues by integrating multiple public scRNA-seq datasets from independent studies for both human (including GSE131778 [12], GSE155512 [13] and GSE155468 [14]) and mouse (including GSE174384 [15], GSE117963 [16], GSE131776 [12] and GSE155513 [13]) using the R package Harmony which has been shown to reduce technical batch effects [17]. Briefly, the indicated datasets were SCTransformed and merged using the merge command of Seurat package [18]. The RunHarmony command was then used to integrate these datasets. UMAP coordinates, neighbors and clusters were then calculated with the reduction parameter set to ‘harmony’. FindAllMarkers function in Seurat was used to identify marker genes for each cluster.

scRNA-seq data of embryonic (Study # SCP1021) and adult (Study # SCP498) human heart [19] including the UMAP coordinate, cell type, gene expression of each cell were obtained from BROAD Institute Single Cell Portal. UMAP visualization of cell cluster and gene expression was generated by custom R scripts. Count matrix of scRNA-seq for embryonic (E10.5) [20] and non-cardiac cells from adult mouse heart [21] were downloaded from GEO (GSE122403) and ArrayExpress database (E-MTAB-6173), respectively, and processed using Seurat package as we previously described [7]. The processed scRNA-seq data of adult mouse heart was obtained from Tabula Muris [22] and visualized using Seurat package [18]. scRNA-seq datasets generated in embryonic human gut (<https://www.gutcellatlas.org/>) [23] and adult human colon (GSE156905) [24], as well as mouse colon and ileum (Single Cell Portal, SCP1038) were re-analyzed as we previously described [25].

**Quantitative reverse transcription-PCR (qRT-PCR) analysis**

Total RNA from mouse tissues was isolated with TRIzol reagent (Invitrogen). 0.8 μg of RNA was used as template for reverse transcription (RT) with random hexamer primers using the High Capacity RNA-to-cDNA kit (Invitrogen). Real time PCR was performed with SYBR green PCR master mix (Applied Biosystems) and respective primers listed in **Online Table S2**. All samples were amplified in duplicate. Relative gene expression was converted using the 2^-🛆🛆CT^ method against the internal control house-keeping gene glyceraldehyde-3-phosphate dehydrogenase (*Gapdh*) where 🛆🛆CT = (CT_experimental gene_ – CT_experimental_ *_Gapdh_*) – (CT_control gene_ – CT_control_ *_Gapdh_*).

**Protein extraction and Western blotting**

Protein lysates were extracted from different mouse tissues or cultured cells by RIPA buffer (Thermo Fisher Scientific) plus 1% protease/phosphatase inhibitor cocktail (Thermo Fisher Scientific). After sonication and centrifugation of the lysates, proteins in the supernatant were quantified by BCA assay (Thermo Fisher Scientific) and resolved on a 7.5% SDS-PAGE gel with 15 μg per lane where appropriate. Primary antibodies against PRDM16 (R&D, AF6295, sheep, 1:1000), ACTA2 (Sigma, A2547, mouse, 1:5000), GAPDH (Santa Cruz Biotechnology, sc-32233, mouse, 1:1000), CNN1 (Calponin 1, Proteintech, 13938-1AP, Rabbit, 1:2000), PCNA (Santa Cruz, sc-56, mouse, 1:1000), and TGFB1I1 (Hic-5, BD, 611164, mouse, 1:2000) and VCL (Sigma, V9264, mouse, 1:2000) were used. Secondary antibodies conjugated with horseradish peroxidase were then used to visualize the target proteins in blot. Images were acquired by Amersham ImageQuant 800 biomolecular imager.

**Mouse breeding and genotyping**

The *Prdm16* flox mice with exon 9 flanked by loxP sites [26] were purchased from the Jackson Laboratory (Stock #: 024992). *Myh11*-CreER^T2^ transgenic mice (originally generated by Dr. Stefan Offermanns’s lab as previously published [27]) were obtained from Dr. Joseph Miano at the University of Rochester (now at Augusta University). Constitutive *Myh11*-Cre line was recently developed by us [28]. All strains have been maintained on a C57BL/6 background. To generate *Prdm16* inducible SM-specific KO (iSM KO) mice, female mice homozygous for *Prdm16* flox allele (*Prdm16^F/F^*) were crossed with male *Myh11*-CreER^T2^ mice. Subsequently the *Myh11*-CreER^T2+^; *Prdm16^F/W^* male mice were bred with *Prdm16^F/W^* female mice to generate *Myh11*-CreER^T2+^; *Prdm16^F/F^* mice and *Myh11*-CreER^T2+^; *Prdm16^W/W^* mice that serve as control. At 8 weeks of age, mice from both groups were intraperitoneally injected with tamoxifen (1 mg/mouse/day) for 10 days with 2 days’ break between the first and second 5 injections, followed by a washout of tamoxifen for 2 weeks. Mice were monitored for additional 60 days daily for signs of phenotypes and then sacrificed for histological and bulk RNA-seq analysis. Only male mice were used here because *Myh11*-CreER^T2^ transgene is located on Y chromosome [27]. To obtain constitutive *Prdm16* SM-specific KO (cSM KO) mice, we first generated *Myh11*-Cre^+/-^*; Prdm16^F/W^* mice, by crossing *Myh11*-Cre^+/-^ male mice with *Prdm16^F/F^* female mice. Then *Myh11*-Cre^+/-^*; Prdm16^F/W^* male mice were bred with *Prdm16^F/F^* female mice to produce *Myh11*-Cre^+/-^*; Prdm16^F/F^* (*Prdm16* cSM KO) mice. Age- and sex-matched *Myh11*-Cre^+/-^*; Prdm16^W/W^* mice were used as controls.

**Sections and Hematoxylin & Eosin (H&E) staining**

Mice were euthanized by an overdose of 4% Isoflurane via inhalation, then systemically perfused with PBS via the left ventricle. Isolated thoracic aortic tissues were fixed with 4% paraformaldehyde in PBS overnight at 4°C, washed 3 times with PBS, then kept in 30% sucrose in PBS overnight at 4°C. Fixed tissues were embedded in optimal cutting temperature compound (OCT) and kept at -80°C untill cryo-sectioning. Sections were cut at 8 μm thickness and HE staining was performed following standard protocol as previously described [7]. HE-stained images were captured using an ECHO REVOLVE microscope. Sections were analyzed blindly by an independent investigator for aortic media layer thickness and lumen area using ImageJ software.

**Transcriptome analysis by bulk RNA-seq**

Aortic tissues of 3 *Prdm16* iSM KO and 3 control mice were isolated and adventitial layer was removed under a stereoscope. Total RNA from the aortic tissues was then extracted using TRIzol reagent (Invitrogen) and subjected to whole transcriptome RNA-seq analysis at the Genome Technology Access Center at Washington University. Sequencing libraries were constructed from purified RNA using RiboErase kit (Kapa Biosystems) according to the manufacturer’s instructions. Libraries were sequenced on a NovaSeq 6000 system (Illumina) using paired-end reads extending 150-bp bases.

Obtained RNA-seq reads were then mapped and quantified to Ensembl release 101 mouse reference genome (mm10) with an Illumina DRAGEN Bio-IT. Raw count for each gene was rounded and the integer count was used for subsequent analysis. Only genes with count >10 in all the samples of at least one group were considered as expressed genes and used for subsequent analysis. Differential expression analysis was performed with R package DEseq2 [11]. Cutoff values of fold change greater than 2 and false discovery rate (FDR) less than 0.05 were considered statistically significant between control and KO groups. Principal component analysis (PCA) and volcano plot was generated using custom R script. GO (Gene Ontology) and KEGG pathway analysis was carried out by Metascape (<http://metascape.org>).

**Integrative analysis of GWAS and bulk RNA-seq**

GWAS-identified risk genes for different cardiovascular and related diseases were obtained from GWAS catalog database (<https://www.ebi.ac.uk/gwas/>). As summarized in **Table S5**, individual GWAS traits were manually curated and grouped into broader disease categories based on clinical relevance, including CAD, chronic obstructive pulmonary disease (COPD), blood pressure (BP)-related disorders, stroke, intima-media thickness (IMT), artery calcification (AC), aneurysm and cardiovascular disease (CVD) Differentially expressed genes in mouse aorta following *Prdm16* deletion were intersected with the obtained gene list to identify *Prdm16*-regulated risk genes of cardiovascular diseases. The overlapping results were visualized with R package GOplot [29].

**De novo analysis of PRDM16 ChIP-seq data**

PRDM16 ChIP-seq peaks generated in C57BL/6 mouse heart were obtained from GEO database (GSE179371) [30]. The peaks were annotated using ChIPseeker package [2] and overlapped with differentially expressed genes identified in the aortic tissues of *Prdm16* iSM KO mice. Raw reads of PRDM16 ChIP-seq for whole heart tissues of wild-type mice were downloaded and aligned against Ensembl mouse reference genome mm10 using Bowtie 2 [31]. The generated Bam files were used for Integrative Genomics Viewer (IGV) visualization.

**Mouse left carotid artery ligation injury, neointima analysis and immunofluorescence staining**

Mouse carotid artery ligation was performed as previously described [7]. Briefly, 6-8-week-old male and female *Prdm16* cSM KO and control mice were anesthetized by isoflurane. A total of 6-7 mice per group were used, which provides sufficient power (~80%) to detect a ~30-40% difference in neointima area at a=0.05 and represents a sample size commonly used for neointimal analysis [32-38]. The left common carotid artery was dissected and completely ligated just proximal to the bifurcation. Skin incision was then closed using a 6-0 Vicryl absorbable suture. The right carotid artery served as an uninjured contralateral control. The left and right carotid arteries were harvested 21 days after injury. Tissues were fixed in 4% PFA at 4 °C overnight and then embedded in paraffin. As previously described [32, 35], the ligated carotid artery was serially sectioned at 5 μm from the ligature toward the aortic arch. For each artery, a reference point beneath the ligation site was identified at which the ligature did not distort the vessel and the elastic laminae remained intact as described previously [35]. Sections located at 100, 200, 300 and 400 μm from the reference point were analyzed for neointima formation. The H&E-stained sections were imaged using a fluorescence microscope (REVOLVE, ECHO) under brightfield to assess tissue morphology, and under the FITC channel to visualize the elastin autofluorescence. The internal and external elastic laminae were traced by adjusting the brightness and contrast of H&E images to enhance elastin visibility. In cases where the staining intensity was too strong to clearly visualize elastic laminae, elastin autofluorescence images were manually overlaid to facilitate the analysis. The areas of intima and media were measured by ImageJ software (NIH). For immunofluorescence staining of ACTA2 and MKI67, sections located at 200 μm from the reference point were analyzed. Three KO samples lacked discernible neointima at this position and therefore sections between 100 and 200 μm from the reference point were analyzed instead. Antigen retrieval was carried out by microwaving to heat at 98°C for 10 minutes in citric acid buffer (10 mM, pH 6.0). After blocking with goat serum (10%, Invitrogen) for 30 minutes, sections were then incubated with primary antibodies (anti-ACTA2: Sigma, A2547, mouse, 1:200; anti-MKI67: Cell Signaling, 12202, rabbit, 1:30) at 4°C overnight and subsequently with appropriate secondary antibodies that are conjugated with different fluorophore for 1 hour at room temperature. Following 3 washes with PBS, sections were then immersed with mounting medium (ProLong Gold anti-fade reagent with DAPI, Invitrogen) to stain nuclei. Images were collected by using a confocal microscope (LSM 780 upright, Zeiss) at the imaging core of Augusta University.

**Mouse VSMC isolation and culture**

VSMCs were isolated from thoracic aorta of 8-week-old *Prdm16* cSM KO or control male mice as we previously described [7]. Briefly, dorsal aortas were dissected and isolated. After carefully removing periadventitial tissues, aortas were minced into small pieces and digested in buffer containing 1mg/mL Collagenase II (Worthington, LS004174), 1 mg/mL Soybean Trypsin Inhibitor (Worthington, LS003570) and 0.554 mg/mL Elastase (Worthington, LS002279). The dispersed cells were placed in 6-well plates in DMEM supplemented with 10% FBS DMEM and maintained in culture. Cells between passages 4-6 were used for experiments after cultures were re-established. For gene expression analysis, equal numbers of control and KO cells were seeded in 6-well plates and cultured in DMEM with 10% FBS. Cells were harvested at 48 hours for qRT-PCR and at 72 hours for Western blotting analysis of proliferative genes and SMC contractile markers.

**PDGF-BB treatment, 5-Ethynyl-2-deoxyuridine (EdU) incorporation assays and flow cytometry**

VSMCs isolated from control and *Prdm16* cSM KO mice were grown to 80-90% confluence and serum-starved with DMEM containing 0.2% FBS overnight. EdU incorporation was performed using the Click-iT^TM^ EdU Alexa Fluor^TM^ 594 Flow Cytometry Assay Kit (Thermo#C10646) according to the manufacturer’s instructions. Briefly, cells were treated with recombinant mouse PDGF-BB (Thermo#315-18-10UG) at 50 ng/mL along with the presence of EdU (10 mM) for 24 hours as described previously [7]. For flow cytometry, cells were washed, detached, and processed into single-cell suspensions, stained with a fixable viability dye (Live/Dead fixable blue dead cell stain kit, Thermo#L23105), and then fixed, and permeabilized following kit instructions. Flow cytometry was performed on a Cytek 5-Laser Aurora Spectral Cytometer and data were analyzed using FlowJo 10.10.0 software.

***Prdm16* knockdown in mouse VSMCs**

siRNA targeting mouse *Prdm16* was obtained from Thermo Fisher (Ref#4390771; siRNA ID: 176968) and a scrambled siRNA (Thermo#4390843) was used as control. Transfection of primary mouse VSMCs were carried out using Lipofectamine RNAiMax Transfection Reagent (Invitrogen#13778) at a final siRNA concentration of 60 nM. Cells were harvested 48 hours after transfection for qRT-PCR analysis.

**Luciferase reporter assays**

The WT mouse *Tgfb2* promoter (mm10, chr1: 186,705,000 – 186,706,517, minus strand) and a deletion construct lacking the putative PRDM16-bound region (mm10, chr1: 186,705,246 – 186,705,758, minus strand) identified by ChIP-seq data in mouse embryonic heart were commercially synthesized and cloned into a mammalian luciferase reporter vector by VectorBuilder. A pcDNA expression plasmid expressing PRDM16 was obtained from Addgene (#15503) and prepared by MaxPrep (Thermo#K0491). 10T1/2 cells, A7r5 and PAC1 cells were transfected using PolyJet^TM^ In Vitro DNA transfection reagent (Signagen Laboratories, SL100688), with PRDM16 expression plasmid or empty pcDNA vector as control, as previously described [7]. Promoter activity was quantified using the Dual-Luciferase Reporter Assay System (Promega#E1960) by measuring firefly luciferase normalized to Renilla luciferase. Three independent transfections were performed and all assays were replicated at least twice.

**Statistical analysis**

GraphPad Prism (version 9.2.0) was used for the statistical analysis. All data are expressed as mean ± SEM of at least 3 independent experiments. Tests used for statistical significance evaluations are specified in figure legends. An unpaired 2-tailed *t* test was used for data involving 2 groups only. Values of P<0.05 were considered statistically significant for qRT-PCR analysis and FDR-adjusted P<0.05 was used as the threshold for statistical significance for bulk RNA-seq analysis.

**References**

[1] Whyte WA, Orlando DA, Hnisz D, Abraham BJ, Lin CY, Kagey MH, et al. Master transcription factors and mediator establish super-enhancers at key cell identity genes. Cell 2013; 153: 307-19.

[2] Yu G, Wang LG,He QY. ChIPseeker: an R/Bioconductor package for ChIP peak annotation, comparison and visualization. Bioinformatics 2015; 31: 2382-3.

[3] Aibar S, Gonzalez-Blas CB, Moerman T, Huynh-Thu VA, Imrichova H, Hulselmans G, et al. SCENIC: single-cell regulatory network inference and clustering. Nat Methods 2017; 14: 1083-1086.

[4] Hall IF, Climent M, Quintavalle M, Farina FM, Schorn T, Zani S, et al. Circ_Lrp6, a Circular RNA Enriched in Vascular Smooth Muscle Cells, Acts as a Sponge Regulating miRNA-145 Function. Circ Res 2019; 124: 498-510.

[5] Li B, Qing T, Zhu J, Wen Z, Yu Y, Fukumura R, et al. A Comprehensive Mouse Transcriptomic BodyMap across 17 Tissues by RNA-seq. Sci Rep 2017; 7: 4200.

[6] Wang Z, Zhao X, Zhao G, Guo Y, Lu H, Mu W, et al. PRDM16 deficiency in vascular smooth muscle cells aggravates abdominal aortic aneurysm. JCI Insight 2023; 8.

[7] Dong K, Shen J, He X, Hu G, Wang L, Osman I, et al. CARMN Is an Evolutionarily Conserved Smooth Muscle Cell-Specific LncRNA That Maintains Contractile Phenotype by Binding Myocardin. Circulation 2021; 144: 1856-1875.

[8] Bolger AM, Lohse M,Usadel B. Trimmomatic: a flexible trimmer for Illumina sequence data. Bioinformatics 2014; 30: 2114-20.

[9] Dobin A, Davis CA, Schlesinger F, Drenkow J, Zaleski C, Jha S, et al. STAR: ultrafast universal RNA-seq aligner. Bioinformatics 2013; 29: 15-21.

[10] Liao Y, Smyth GK,Shi W. featureCounts: an efficient general purpose program for assigning sequence reads to genomic features. Bioinformatics 2014; 30: 923-30.

[11] Love MI, Huber W,Anders S. Moderated estimation of fold change and dispersion for RNA-seq data with DESeq2. Genome Biol 2014; 15: 550.

[12] Wirka RC, Wagh D, Paik DT, Pjanic M, Nguyen T, Miller CL, et al. Atheroprotective roles of smooth muscle cell phenotypic modulation and the TCF21 disease gene as revealed by single-cell analysis. Nat Med 2019; 25: 1280-1289.

[13] Pan H, Xue C, Auerbach BJ, Fan J, Bashore AC, Cui J, et al. Single-Cell Genomics Reveals a Novel Cell State During Smooth Muscle Cell Phenotypic Switching and Potential Therapeutic Targets for Atherosclerosis in Mouse and Human. Circulation 2020; 142: 2060-2075.

[14] Li Y, Ren P, Dawson A, Vasquez HG, Ageedi W, Zhang C, et al. Single-Cell Transcriptome Analysis Reveals Dynamic Cell Populations and Differential Gene Expression Patterns in Control and Aneurysmal Human Aortic Tissue. Circulation 2020; 142: 1374-1388.

[15] Kalluri AS, Vellarikkal SK, Edelman ER, Nguyen L, Subramanian A, Ellinor PT, et al. Single-Cell Analysis of the Normal Mouse Aorta Reveals Functionally Distinct Endothelial Cell Populations. Circulation 2019; 140: 147-163.

[16] Dobnikar L, Taylor AL, Chappell J, Oldach P, Harman JL, Oerton E, et al. Disease-relevant transcriptional signatures identified in individual smooth muscle cells from healthy mouse vessels. Nat Commun 2018; 9: 4567.

[17] Korsunsky I, Millard N, Fan J, Slowikowski K, Zhang F, Wei K, et al. Fast, sensitive and accurate integration of single-cell data with Harmony. Nat Methods 2019; 16: 1289-1296.

[18] Hao Y, Hao S, Andersen-Nissen E, Mauck WM, 3rd, Zheng S, Butler A, et al. Integrated analysis of multimodal single-cell data. Cell 2021; 184: 3573-3587 e29.

[19] Tucker NR, Chaffin M, Fleming SJ, Hall AW, Parsons VA, Bedi KC, Jr., et al. Transcriptional and Cellular Diversity of the Human Heart. Circulation 2020; 142: 466-482.

[20] Li G, Tian L, Goodyer W, Kort EJ, Buikema JW, Xu A, et al. Single cell expression analysis reveals anatomical and cell cycle-dependent transcriptional shifts during heart development. Development 2019; 146.

[21] Skelly DA, Squiers GT, McLellan MA, Bolisetty MT, Robson P, Rosenthal NA, et al. Single-Cell Transcriptional Profiling Reveals Cellular Diversity and Intercommunication in the Mouse Heart. Cell Rep 2018; 22: 600-610.

[22] Tabula Muris C, Overall c, Logistical c, Organ c, processing, Library p, et al. Single-cell transcriptomics of 20 mouse organs creates a Tabula Muris. Nature 2018; 562: 367-372.

[23] Adachi Y, Ueda K, Nomura S, Ito K, Katoh M, Katagiri M, et al. Beiging of perivascular adipose tissue regulates its inflammation and vascular remodeling. Nat Commun 2022; 13: 5117.

[24] Wright CM, Schneider S, Smith-Edwards KM, Mafra F, Leembruggen AJL, Gonzalez MV, et al. scRNA-Seq Reveals New Enteric Nervous System Roles for GDNF, NRTN, and TBX3. Cell Mol Gastroenterol Hepatol 2021; 11: 1548-1592 e1.

[25] He X, Dong K, Shen J, Hu G, Mintz JD, Atawia RT, et al. The LncRNA Carmn is a Critical Regulator for Gastrointestinal Smooth Muscle Contractile Function and Motility. bioRxiv 2022; 2022.06. 28.498024.

[26] Cohen P, Levy JD, Zhang Y, Frontini A, Kolodin DP, Svensson KJ, et al. Ablation of PRDM16 and beige adipose causes metabolic dysfunction and a subcutaneous to visceral fat switch. Cell 2014; 156: 304-16.

[27] Wirth A, Benyo Z, Lukasova M, Leutgeb B, Wettschureck N, Gorbey S, et al. G12-G13-LARG-mediated signaling in vascular smooth muscle is required for salt-induced hypertension. Nat Med 2008; 14: 64-8.

[28] Dong K, Bai Z, He X, Zhang L, Hu G, Yao Y, et al. Generation of a novel constitutive smooth muscle cell-specific Myh11-driven Cre mouse model. J Mol Cell Cardiol 2025; 202: 144-152.

[29] Walter W, Sanchez-Cabo F,Ricote M. GOplot: an R package for visually combining expression data with functional analysis. Bioinformatics 2015; 31: 2912-4.

[30] Wu T, Liang Z, Zhang Z, Liu C, Zhang L, Gu Y, et al. PRDM16 Is a Compact Myocardium-Enriched Transcription Factor Required to Maintain Compact Myocardial Cardiomyocyte Identity in Left Ventricle. Circulation 2022; 145: 586-602.

[31] Langmead B,Salzberg SL. Fast gapped-read alignment with Bowtie 2. Nat Methods 2012; 9: 357-9.

[32] Ma Q, Yang Q, Xu J, Zhang X, Kim D, Liu Z, et al. ATIC-Associated De Novo Purine Synthesis Is Critically Involved in Proliferative Arterial Disease. Circulation 2022; 146: 1444-1460.

[33] Wang Z, Cai M, Tay LWR, Zhang F, Wu P, Huynh A, et al. Phosphatidic acid generated by PLD2 promotes the plasma membrane recruitment of IQGAP1 and neointima formation. FASEB J 2019; 33: 6713-6725.

[34] Hu W, Wu X, Jin Z, Wang Z, Guo Q, Chen Z, et al. Andrographolide Promotes Interaction Between Endothelin-Dependent EDNRA/EDNRB and Myocardin-SRF to Regulate Pathological Vascular Remodeling. Front Cardiovasc Med 2021; 8: 783872.

[35] de Waard V, Arkenbout EK, Carmeliet P, Lindner V,Pannekoek H. Plasminogen activator inhibitor 1 and vitronectin protect against stenosis in a murine carotid artery ligation model. Arterioscler Thromb Vasc Biol 2002; 22: 1978-83.

[36] Zhang W, Pan L, Wu X, Slivano OJ, Dong K,Long X. Functional characterization of human IL-8 in vascular stenosis using a novel humanized transgenic mouse model. Vascul Pharmacol 2024; 157: 107438.

[37] Kirabo A, Oh SP, Kasahara H, Wagner KU,Sayeski PP. Vascular smooth muscle Jak2 deletion prevents angiotensin II-mediated neointima formation following injury in mice. J Mol Cell Cardiol 2011; 50: 1026-34.

[38] Gong H, Ni J, Xu Z, Huang J, Zhang J, Huang Y, et al. Shp2 in myocytes is essential for cardiovascular and neointima development. J Mol Cell Cardiol 2019; 137: 71-81.

**Online Table S1**

**Information of H3K27ac ChIP-seq data of human arterial tissues in ENCODE used for identification of artery super-enhancers.**

| *Tissue* | *Sample ID* | *ChIP-seq group* | *ENCODE ID* | |
| --- | --- | --- | --- | --- |
|  |  |  | **Bam file** | **narrowPeak file** |
| Aorta | Aorta 1 | H3K27ac | ENCFF265HDL | ENCFF072EQH |
|  |  | Control | ENCFF861FUK |  |
|  | Aorta 2 | H3K27ac | ENCFF434DCE | ENCFF064PQH |
|  |  | Control | ENCFF278NFU |  |
| Ascending aorta | As. Aorta 1 | H3K27ac | ENCFF823SXL | ENCFF020COG |
|  |  | Control | ENCFF893ZJC |  |
|  | As. Aorta 2 | H3K27ac | ENCFF128VGR | ENCFF208DZK |
|  |  | Control | ENCFF299AOC |  |
| Tibial artery | T. Artery 1 | H3K27ac | ENCFF140QKE | ENCFF134QRV |
|  |  | Control | ENCFF259JSG |  |
|  | T. Artery 2.1 | H3K27ac | ENCFF437QEP | ENCFF121HDP |
|  | T. Artery 2.2 | H3K27ac | ENCFF459ELZ |  |
|  |  | Control | ENCFF400VJM |  |
| Thoracic aorta | Th. Aorta 1 | H3K27ac | ENCFF946NGS | ENCFF595RQJ |
|  |  | Control | ENCFF901HZC |  |
|  | Th. Aorta 2 | H3K27ac | ENCFF816GNU | ENCFF121HDP |
|  |  | Control | ENCFF924RQL |  |

**Online Table S2**

**List of primers used for qRT-PCR (F: forward; R: reverse).**

| *Gene name* | *Primer name* | *Sequence (5’-3’)* | *Application* |
| --- | --- | --- | --- |
| *Prdm16* | P1 (F) | TAGTGTGTAGCTGCTTCTGGGCTCA | qRT-PCR, detection of exon 1/2 |
|  | P2 (R) | ACAGGATGCCGTCTTCGGTCTCCT |  |
|  | P9 (F) | TGCCTAAGGTGTGCCCAGCACAGC | qRT-PCR, detection of exon 9/10 |
|  | P10 (R) | CGCAGGTACTTCTCTTTCAGGACTC |  |
| *Tgfb2* | F | TTGTTGCCCTCCTACAGACTGG | qRT-PCR |
|  | R | GTAAAGAGGGCGAAGGCAGCAA |  |
| *Adamts8* | F | TCATGCAACACAGAGGAATGTCCAC | qRT-PCR |
|  | R | CTCTGCAAAACAGCTTGCATCGGTC |  |
| *Adamts14* | F | TTCCACAGGTTCCACTGGTCTCGCT | qRT-PCR |
|  | R | GCATTGCTCATCCATGGAGTAGTCG |  |
| *Col14a1* | F | GAGGTTCAACTTCAGGCTTGTGCGC | qRT-PCR |
|  | R | GGCATTCAAGTGCCACTCTATTCTG |  |
| *Hgf* | F | GACGGTATCCATCACTAAGAGTGGC | qRT-PCR |
|  | R | CTTCTTCCCCTCGAGGATTTCGACA |  |
| *Adamtsl3* | F | TTTGCGGAGATGTCTGACTGG | qRT-PCR |
|  | R | ACTGCACGTCATTGTAGGCTG |  |
| *Ccn3* | F | AGATGAGACCCTGTGACCAGAGCAG | qRT-PCR |
|  | R | GCAGAAGTACTGACAGTTCGGCTCA |  |
| *Col3a1* | F | CCTGGCTCAAATGGCTCAC | qRT-PCR |
|  | R | CAGGACTGCCGTTATTCCCG |  |
| *Dusp1* | F | CTACCAGTACAAGAGCATCCCTGTG | qRT-PCR |
|  | R | CTCATGAGGTAAGCAAGGCAGATGG |  |
| *Fos* | F | CGGGTTTCAACGCCGACTA | qRT-PCR |
|  | R | TGGCACTAGAGACGGACAGAT |  |
| *Id1* | F | CTGCTCTACGACATGAACGGCTGCT | qRT-PCR |
|  | R | TCAGCGACACAAGATGCGATCGTCG |  |
| *Pcna* | F | TTGCACGTATATGCCGAGACC | qRT-PCR |
|  | R | GGTGAACAGGCTCATTCATCTCT |  |
| *Mki67* | F | CAAGGCGAGCCTCAAGAGATA | qRT-PCR |
|  | R | TGTGCTGTTCTACATGCCCTG |  |
| *Lmod1* | F | CAGAGGAATCAAACGGACAAACAG | qRT-PCR |
|  | R | GCTTGCTTTCATCCACAGACATC |  |
| *Acta2* | F | ATGCTCCCAGGGCTGTTTTCCCAT | qRT-PCR |
|  | R | GTGGTGCCAGATCTTTTCCATGTCG |  |
| *Cnn1* | F | TCATCTGCACCTCTGCTTTG | qRT-PCR |
|  | R | GGGCCAGCTTGTTCTTTACT |  |
| *Tagln* | F | TGACATGTTCCAGACTGTTGACCTCT | qRT-PCR |
|  | R | CTTCATAAACCAGTTGGGATCTCCAC |  |
| *Ccnd3* | F | ACACCTTTGCGATGTATCCTCC | qRT-PCR |
|  | R | GAGGGCAGCTTCGATCTGTTC |  |
| *Gapdh* | F | GGCATTGCTCTCAATGACAA | Internal control for qRT-PCR analysis |
|  | R | TGTGAGGGAGATGCTCAGTG |  |

Notes: location of primer P1, P2, P9 and P10 for *Prdm16* gene is illustrated in Online Figure S6A. All the primers are designed for mouse genes.

**Online Table S3**

**List of primers used for genotyping (F: forward; R: reverse).**

| *Primer name* | *Sequence (5’-3’)* |
| --- | --- |
| F | CATGGTTCACATGGTCAAGACCAC |
| R1 | CACAGTCCTTGCACTTGATCTGCGT |
| R2 | AGAGCTGCAGGGAGATTGACAAGTG |

Notes: location of primer F, R1 and R2 was illustrated in Online Figure S6A.

**Online Table S5**

**GWAS Catalog traits and their corresponding categories used for integrative analysis**

| **Original GWAS traits (GWAS Catalog database)** | **Disease Category (This study)** |
| --- | --- |
| Abdominal Aortic Aneurysm | Aneurysm |
| aortic aneurysm | Aneurysm |
| coronary aneurysm | Aneurysm |
| coronary artery calcification | Artery calcification |
| carotid artery intima media thickness | Intima-media thickness |
| common carotid intimal medial thickness | Intima-media thickness |
| blood pressure | Blood pressure-related disorders |
| diastolic blood pressure | Blood pressure-related disorders |
| systolic blood pressure | Blood pressure-related disorders |
| essential hypertension | Blood pressure-related disorders |
| hypertension | Blood pressure-related disorders |
| mean arterial pressure | Blood pressure-related disorders |
| treatment-resistant hypertension | Blood pressure-related disorders |
| acute myocardial infarction | Cardiovascular disease |
| atrial fibrillation | Cardiovascular disease |
| myocardial infarction | Cardiovascular disease |
| resting heart rate | Cardiovascular disease |
| Cardiovascular disease | Cardiovascular disease |
| coronary atherosclerosis measurement | Coronary artery disease |
| coronary artery disease | Coronary artery disease |
| carotid atherosclerosis | Coronary artery disease |
| chronic obstructive pulmonary disease | Chronic obstructive pulmonary disease |
| stroke | Stroke |
| Ischemic stroke | Stroke |
| large artery stroke | Stroke |
| small vessel stroke | Stroke |


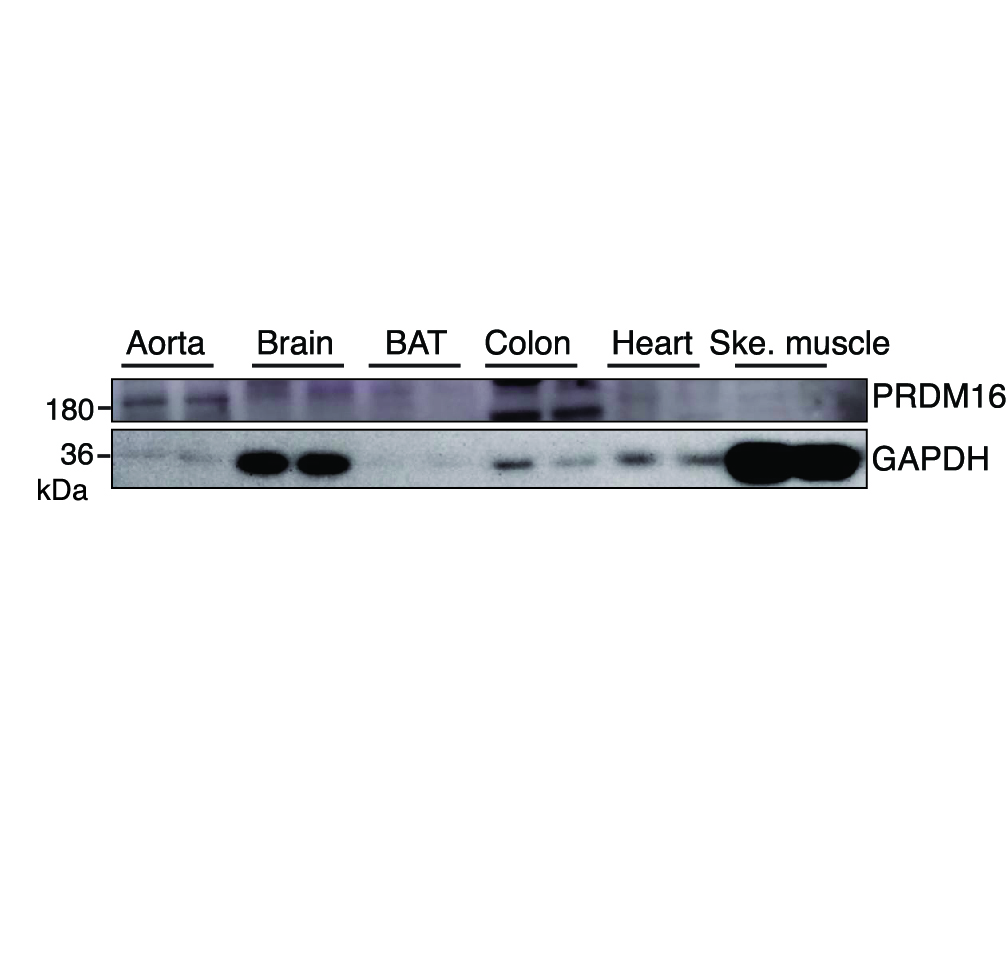


**Online Figure S1. Independent replicate Western blots of PRDM16 across tissues.**

**
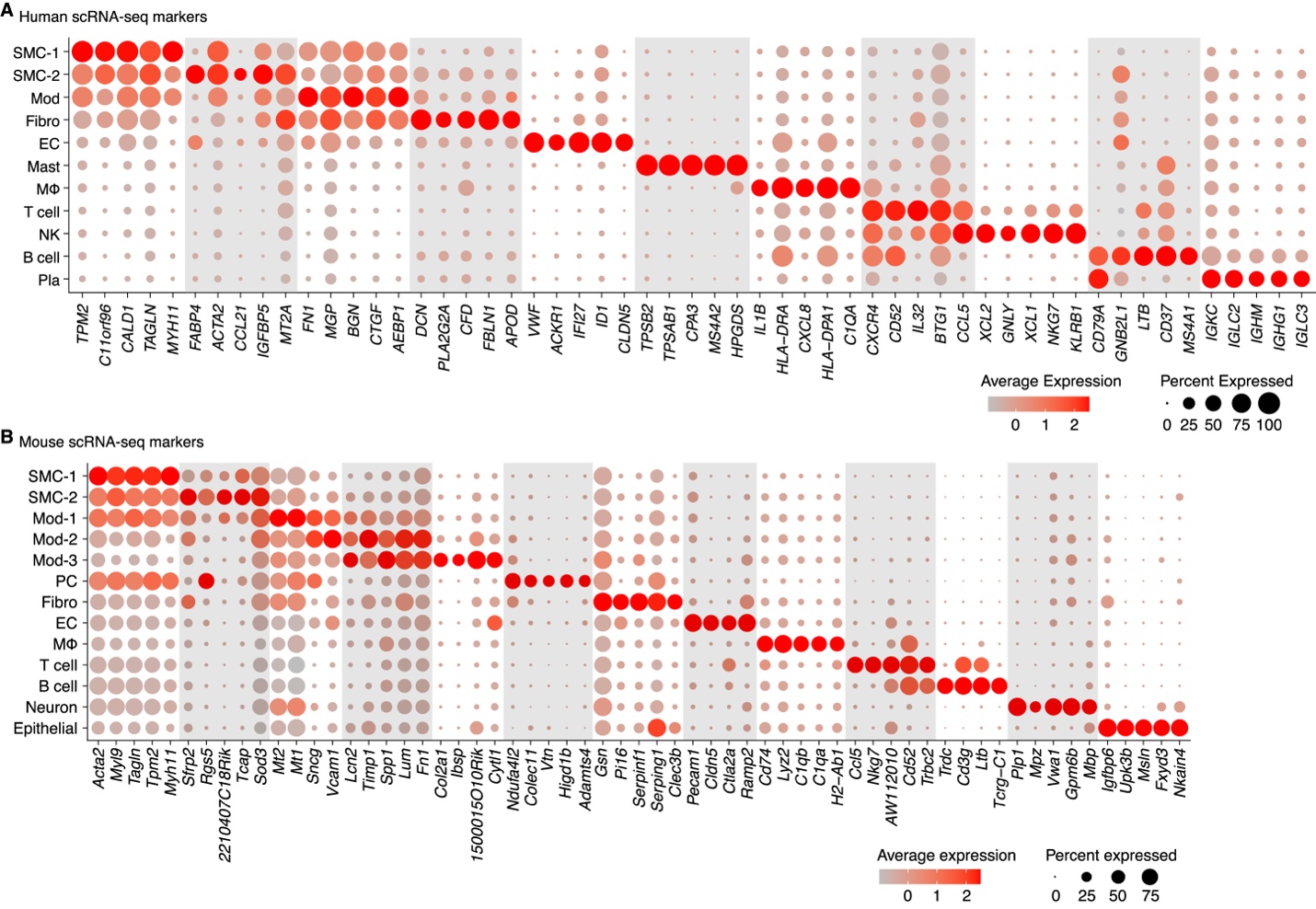
**

**Online Figure S2. Dot plot showing the top 5 genes defining each cell cluster for the merged scRNA-seq data of (A) human arterial tissues (GSE131778, GSE155512, GSE155468), and (B) mouse normal and atherosclerotic arterial tissues (GSE174384, GSE117963, GSE131776, GSE155513).** Mod: modulated SMCs; Fibro: fibroblast; EC: endothelial cell; MΦ: macrophage; NK: natural killer cell; Pla: plasma cell.

**
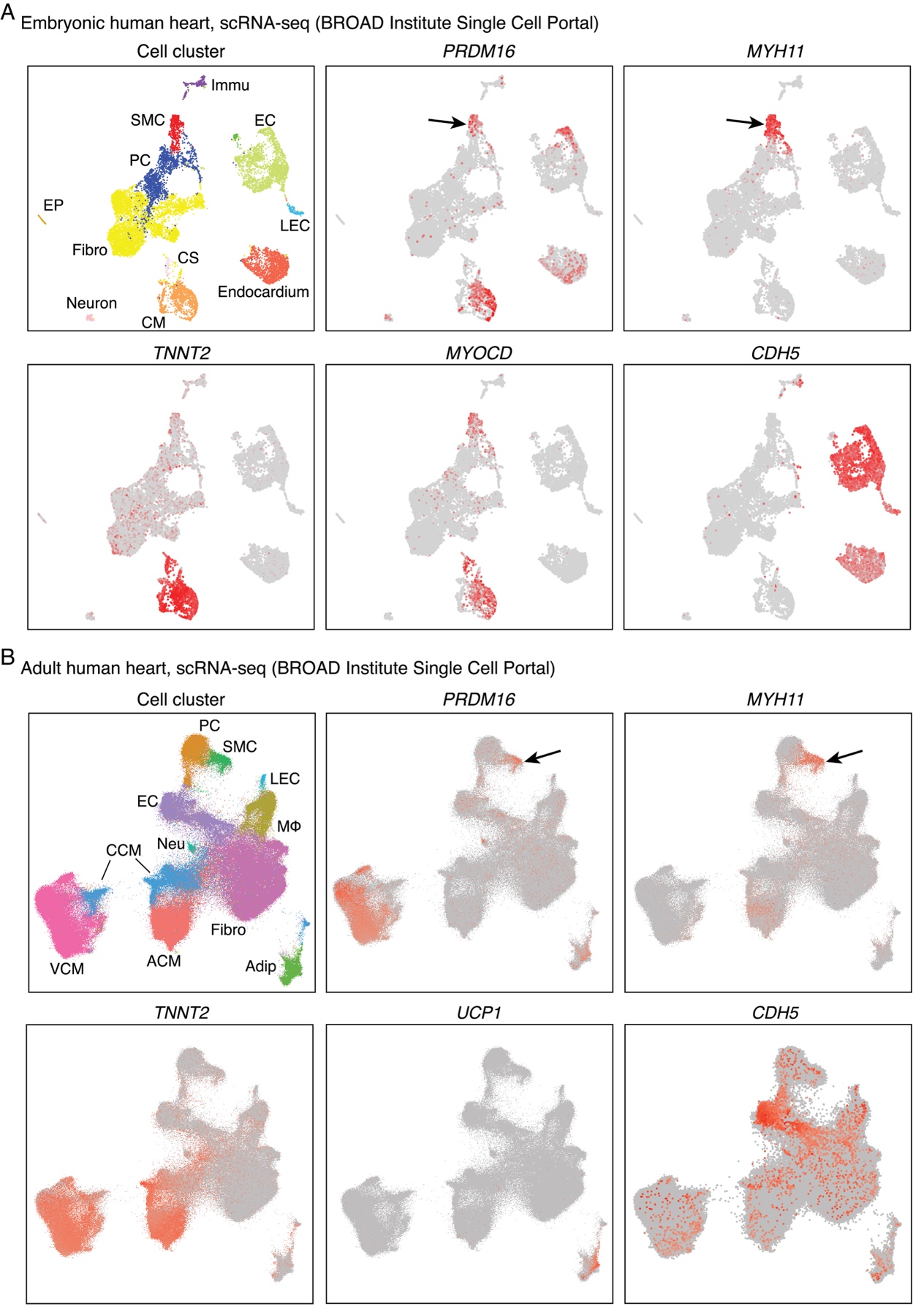
**

**Online Figure S3. PRDM16 expression in embryonic (A) and adult human heart (B) as revealed by scRNA-seq analysis.** EC: endothelial cell; LEC: lymphatic endothelial cell; PC: pericyte; CS: conduction system; CM: cardiomyocyte; EP: epicardium; Immu: immune cells; Fibro: fibroblast; MΦ: macrophage; VCM: ventricular cardiomyocyte; ACM: atrial cardiomyocyte; CCM: cytoplasmic cardiomyocyte; Neu: neuronal cell; Adip: adipocyte. *MYH11*, *TNNT2*, *CDH5* and *UCP1* are used as markers for SMCs, CMs, ECs and adipocytes, respectively. *MYOCD* is used as markers for both SMCs and CMs. Both datasets were downloaded from BROAD Institute Single Cell Portal (links are provided in the Methods) and original annotation of cell types are used. Arrows point to SMC cluster.

**
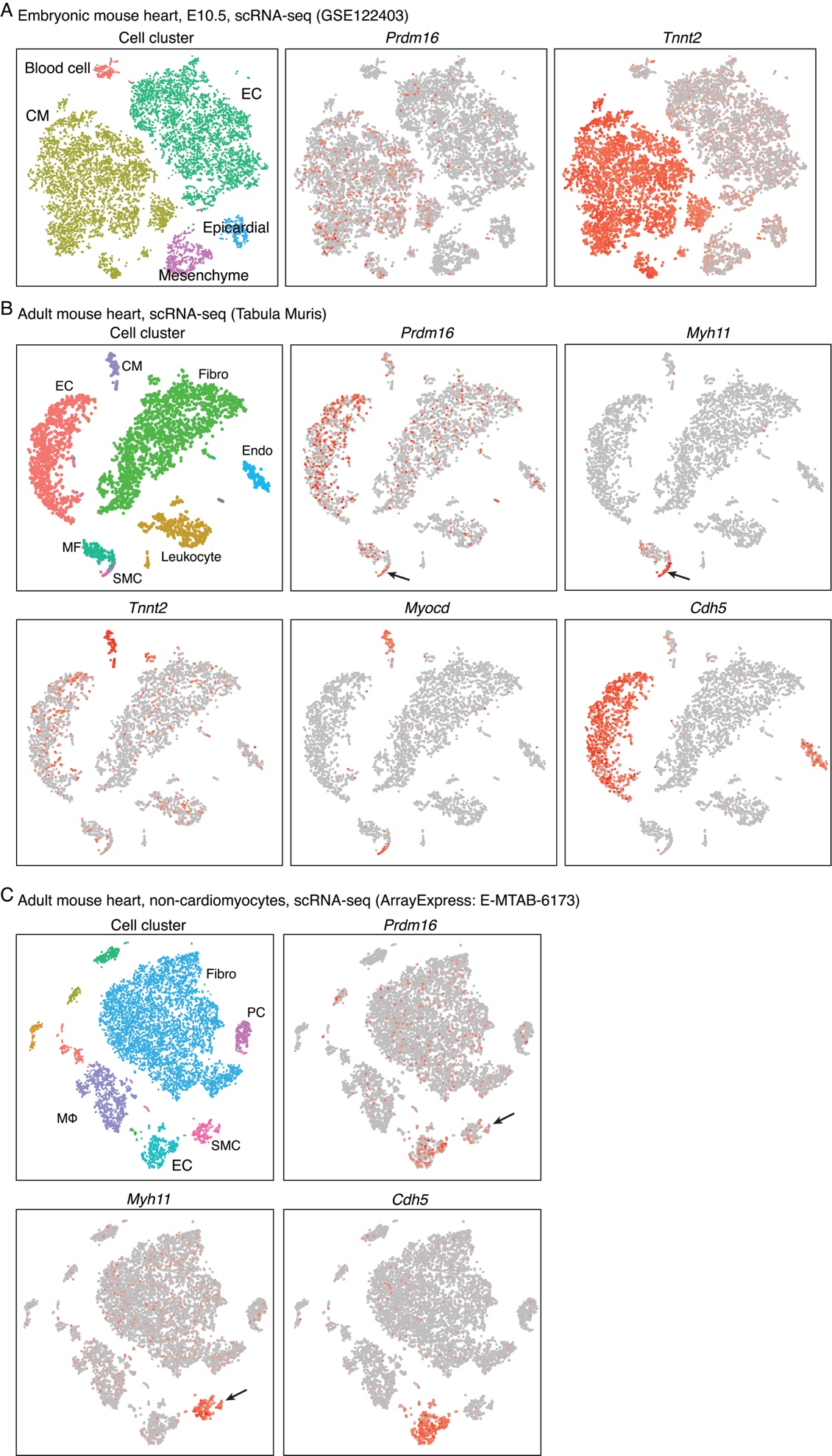
**

**Online Figure S4. *Prdm16* expression in embryonic (A) and adult mouse heart (B-C) as revealed by scRNA-seq analysis.** EC: endothelial cell; CM: cardiomyocyte; Fibro: fibroblast; MΦ: macrophage; MF: myofibroblast cell; Endo: endocardial cell; PC: pericyte. *Myh11*, *Tnnt2* and *Cdh5* were used as markers for SMCs, CMs and ECs, respectively. *Myocd* was used as a marker for both SMCs and CMs. The original annotation for cell types in adult heart from Tabula Muris database was used. Arrows point to SMC cluster.


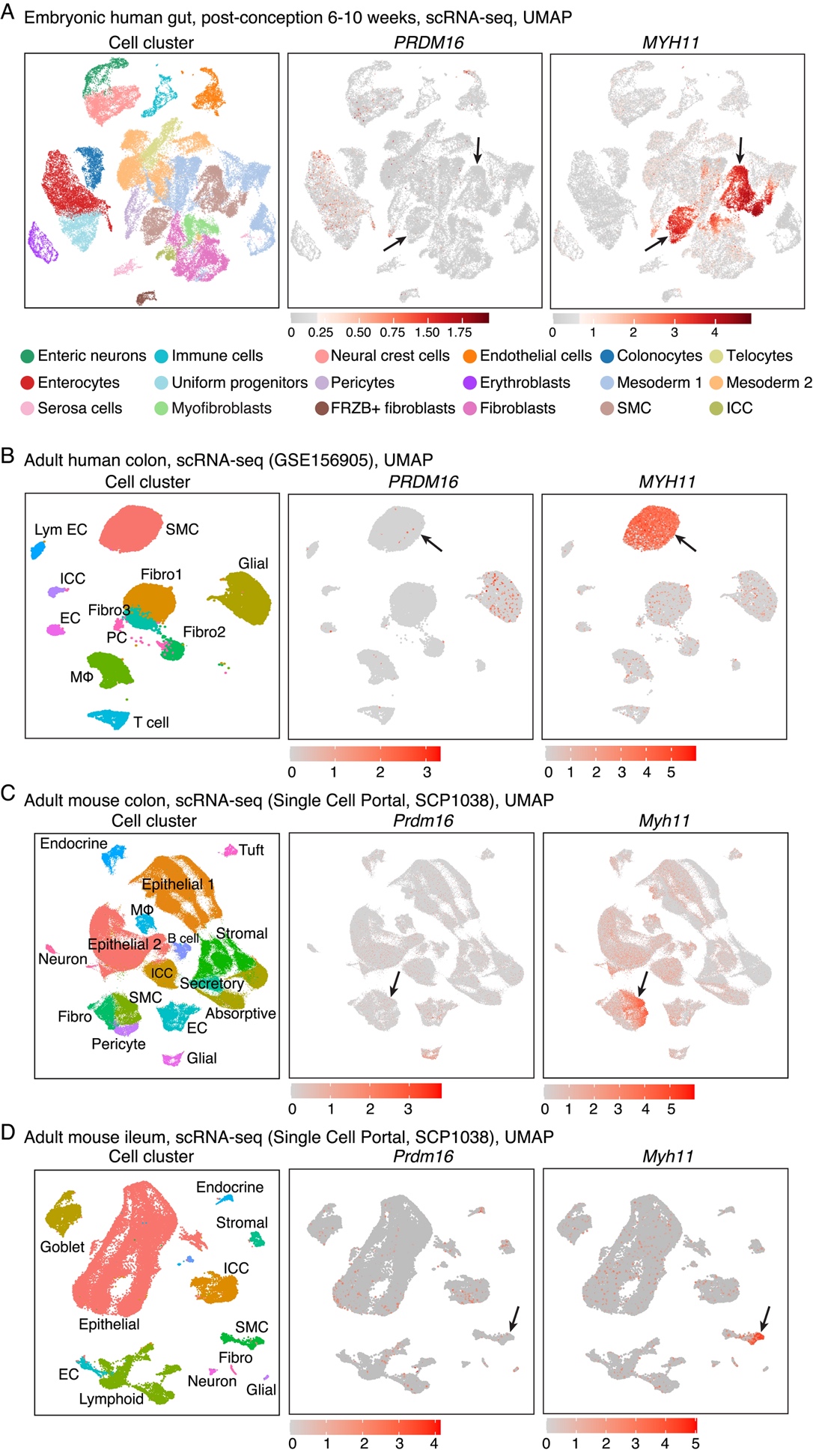


**Online Figure S5. *PRDM16* expression in human and mouse gastrointestinal tissues as revealed by scRNA-seq. (A)** *PRDM16* expression revealed by scRNA-seq of embryonic human gut (post-conception 6-10 weeks), **(B)** adult human colon, **(C)** adult mouse colon and **(D)** ileum tissues. ICC: interstitial cells of Cajal; PC: pericyte; Lym EC: Lymphatic endothelial cell; Fibro: Fibroblast; MΦ: Macrophage. Arrows point to SMC clusters.


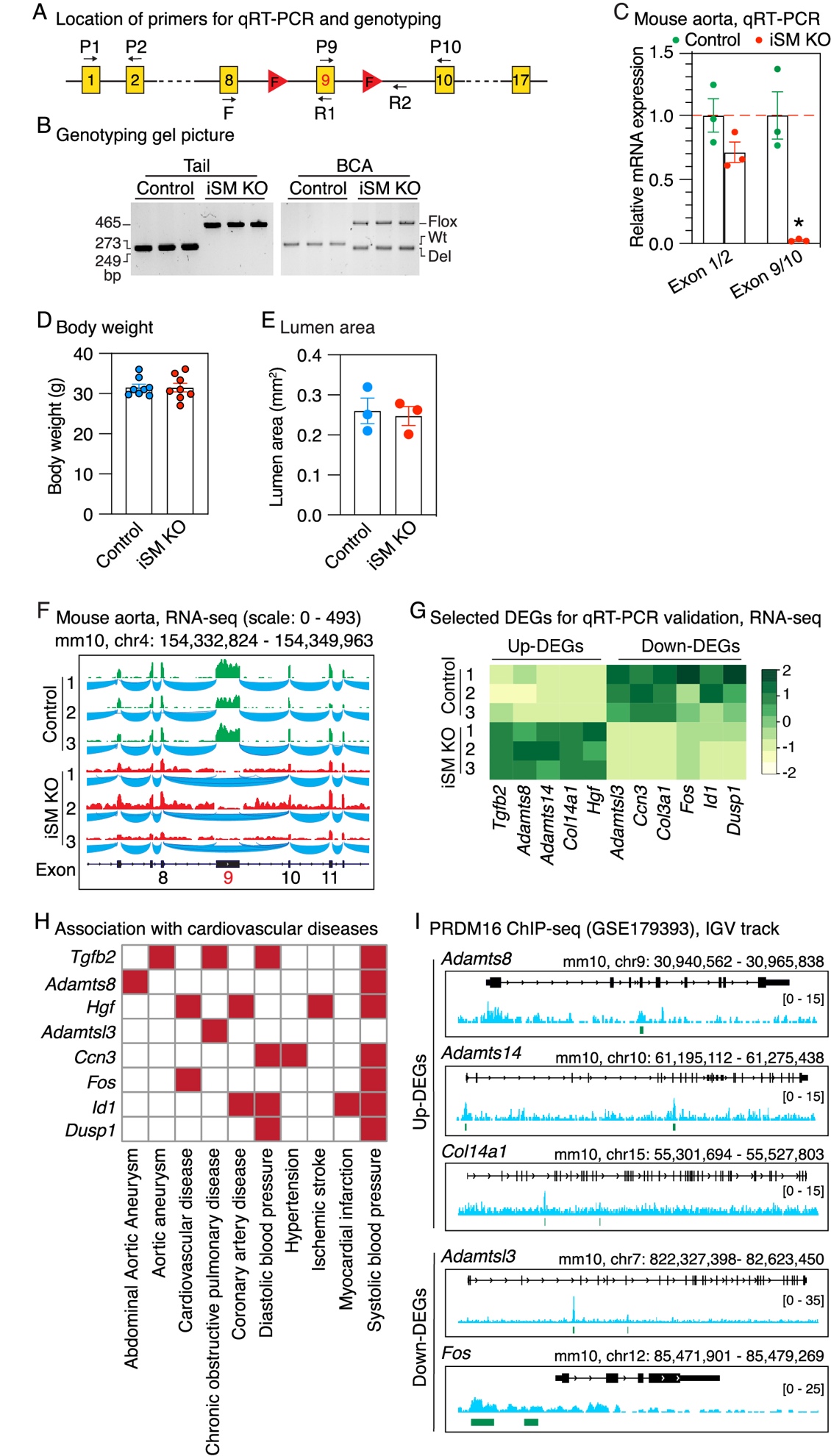


**Online Figure S6. Histological and bulk RNA-seq analyses of aortic tissues from *Prdm16* inducible SMC-specific knockout (iSM KO) mice. (A)** Schematic diagram illustrating the location of primers used for *Prdm16* gene qRT-PCR (P1/P2 for detecting exon 1 and 2, P9 and P10 for detecting exon 9 and 10, respectively) and genotyping (F, R1 and R2). **(B)** Representative agarose gel picture of PCR genotyping using DNA extracted from tail and brachiocephalic artery (BCA) as the template. **(C)** qRT-PCR analysis of *Prdm16* expression in aortic tissues of *Prdm16* iSM KO and control mice, using primers across exon 1 and 2, as well as exon 9 and 10 (PCR amplicon is depicted in **“A”**), respectively. Error bars represent mean ± SEM. N=3 for both control and KO mice; *P<0.05; Unpaired Student’s *t* test. **(D)** Body weight of *Prdm16* iSM KO mice and control mice prior to sacrifice. N=8 for both control and KO mice. Error bars represent mean ± SEM. **(E)** Quantification of lumen area for aortic sections of *Prdm16* iSM KO mice and control mice. N=3 for both control and KO group. Error bars represent mean ± SEM. **(F)** Distribution of RNA-seq junction reads surrounding *Prdm16* exon 9 showing dramatic reduction in number of junction reads spanning *Prdm16* exon 8-9, and exon 9-10 in *Prdm16* iSM KO mice as compared to control mice. **(G)** Heatmap showing the up- and down-DEGs that were identified by RNA-seq and selected for qRT-PCR validation. **(H)** Heatmap showing association of the indicated genes with cardiovascular diseases identified by GWAS studies. **(I)** Integrative Genomics Viewer (IGV) visualization of PRDM16 occupancy at selected target gene loci. Green bars indicate the significant PRDM16 ChIP-seq peaks identified by the original study.

Note: All the mice are male mice due to the inducible *Myh11*-CreER^T2^ driver is Y chromosome-linked and restricted to male mice.


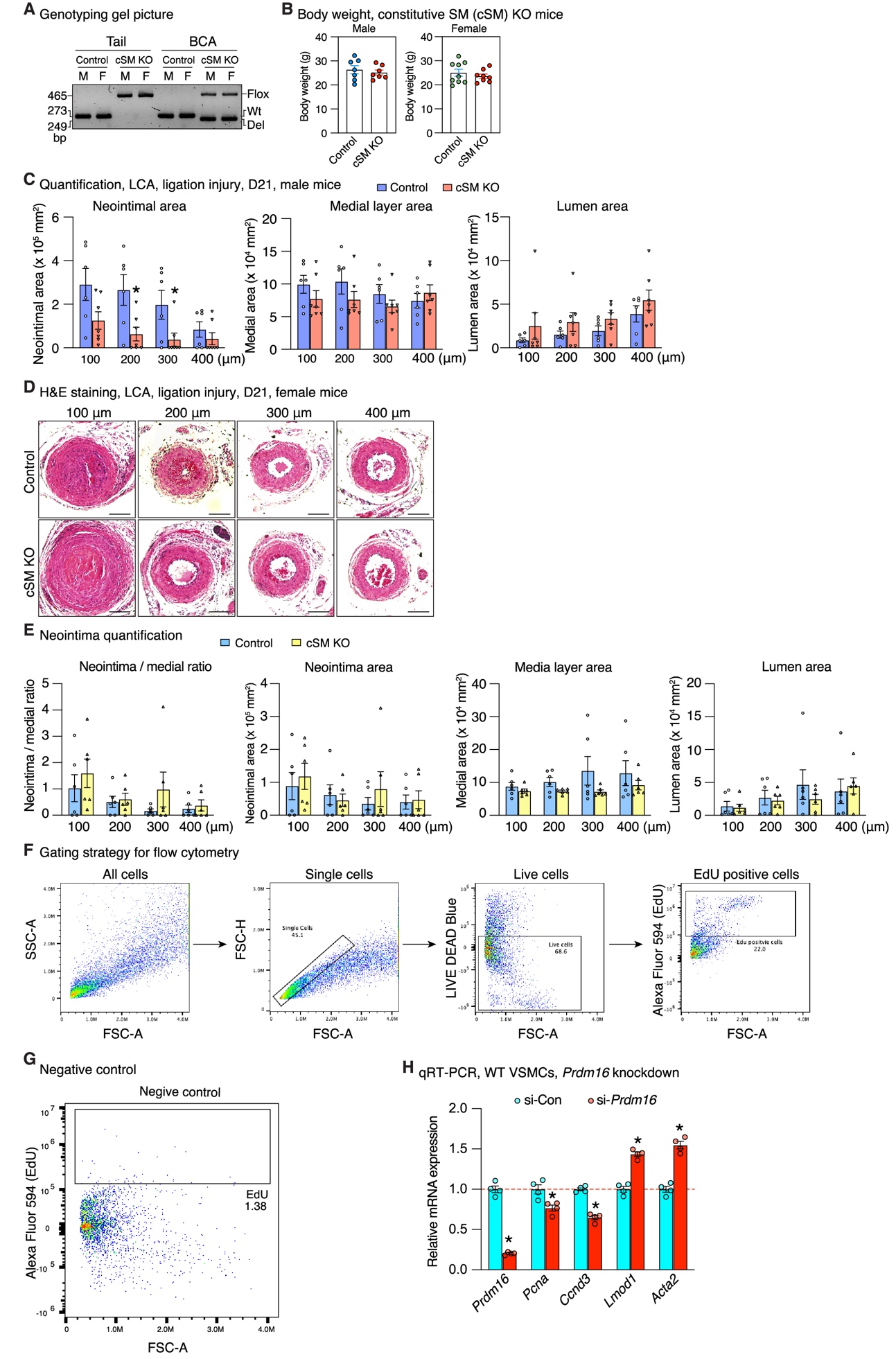


**Online Figure S7. Analysis of ligation-induced neointima formation in the left carotid artery of *Prdm16* constitutive SMC-specific KO (cSM KO) mice. (A)** Representative agarose gel picture of PCR genotyping using DNA extracted from tail and brachiocephalic artery (BCA) as the template. M: male; F: female. **(B)** Body weight of the male (left) and female (right) *Prdm16* cSM KO and control mice at approximately 2 months of age. Error bars represent mean ± SEM. For male mice, N=7 for both control and KO group. For female mice, N=9 for control and N=8 for KO group. **(C)** Quantification of neointimal area (left), medial layer area (middle) and lumen area (right) at four positions from the reference point in the injured left carotid artery (LCA) of male mice. Error bars represent mean ± SEM. N=6 for control and N=7 for KO group; *P<0.05; Unpaired Student’s *t* test. **(D)** Hematoxylin & eosin (H&E) staining of LCA of control and constitutive SM *Prdm16* KO female mice 21 days (D21) post ligation injury. Scale bar: 100 μm. **(E)** Quantification of the neointima-to-media layer ratio, neointimal area, medial layer area and lumen area at four positions from the reference point in the injured LCA of female mice. Error bars represent mean ± SEM. N=6 for both control and KO group. **(F)** Gating strategy for flow cytometry analysis. **(G)** Representative flow plot of EdU^+^ cells from a negative control sample without EdU incubation. **(H)** qRT-PCR analysis of VSMCs isolated from WT mice 48 hours after siRNA-mediated knockdown of *Prdm16* (si-*Prdm16*) or scrambled control (si-Con). Error bars represent mean ± SEM. N=4 for both control and *Prdm16* knockdown group; *P<0.05; unpaired Student’s *t* test.


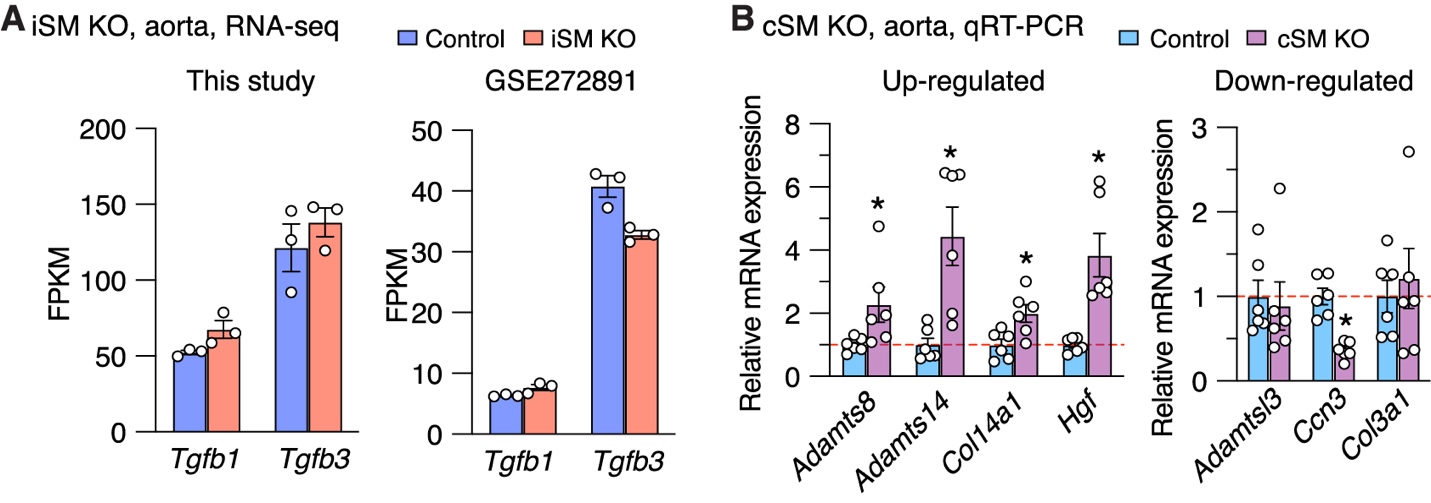


**Online Figure S8. *Tgfb1* and *Tgfb3* expression in aortas of *Prdm16* SMC-specific KO mice. (A)** *Tgfb1* and *Tgfb3* expression in aortas from *Prdm16* inducible SMC-specific KO (iSM KO) mice by bulk RNA-seq analysis from this study (left) and a previous study (right). Error bars represent mean ± SEM. N=3 for both control and KO group. **(B)** qRT-PCR analysis of selected dysregulated genes in aortas of *Prdm16* constitutive SMC-specific KO (cSM KO) and control mice. These dysregulated genes were identified by RNA-seq in *Prdm16* iSM KO mice. Error bars represent mean ± SEM. N=6 for both control and KO group; *P<0.05; unpaired Student’s *t* test.


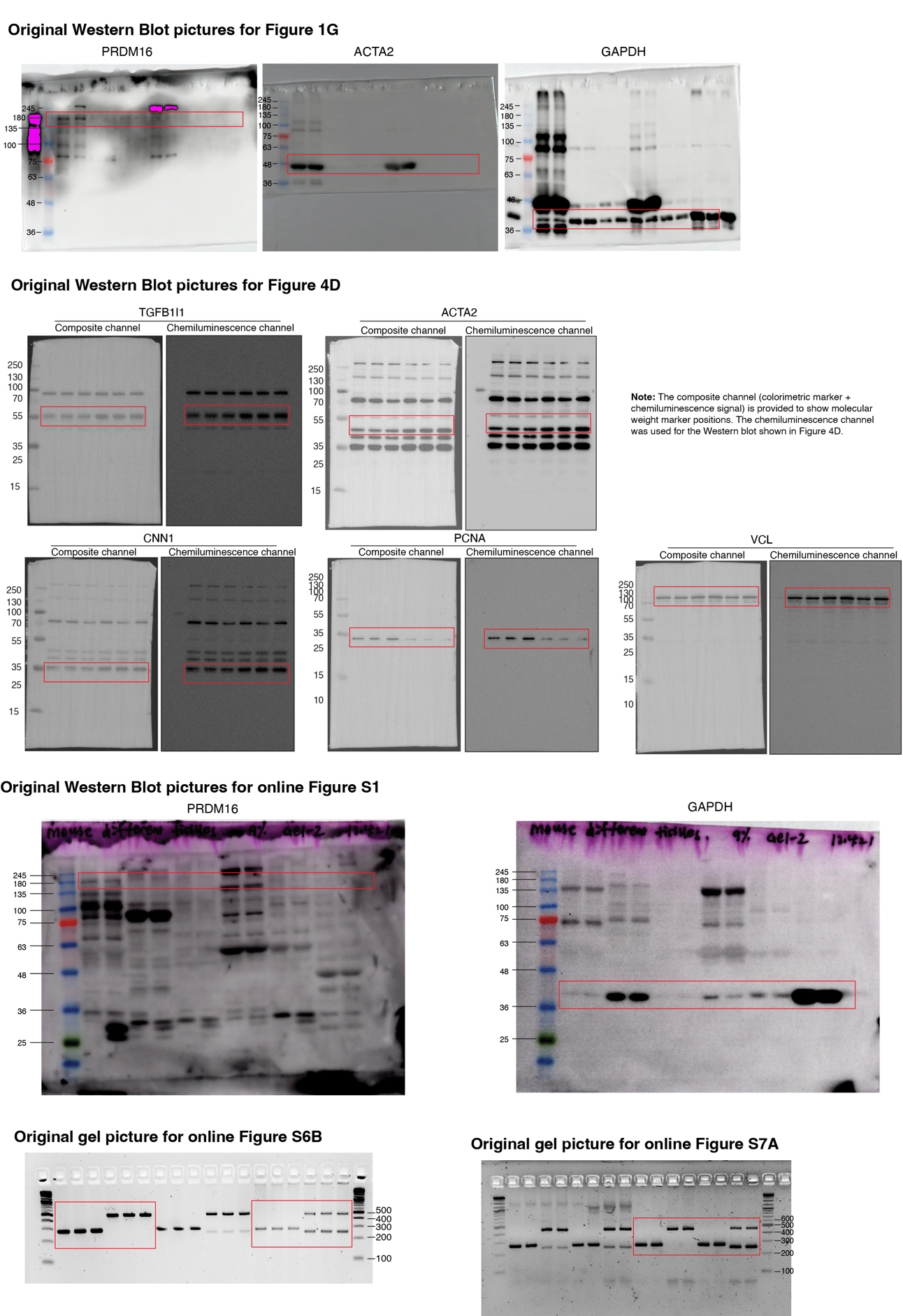


**Online Figure S9. Uncropped original Western blots and gel pictures**
